# Non-Invasive Brain Stimulation for Core Symptoms of Chronic Primary Pain: A Meta-Analysis of RCTs

**DOI:** 10.3390/brainsci16070663

**Published:** 2026-06-24

**Authors:** Alessandra Telesca, Alessandra Vergallito, Anna Vedani, Gaia Locatelli, Benedetta Visiello, Leonor J. Romero Lauro

**Affiliations:** 1 Neuroalgology Unit, Department of Clinical Neuroscience, Fondazione IRCCS Istituto Neurologico Carlo Besta, 20133 Milan, Italy; alessandra.telesca@istituto-besta.it; 2 Neurointensive Care Unit, Department of Neurosurgery, Fondazione IRCCS Istituto Neurologico Carlo Besta, 20133 Milan, Italy; 3 Department of Theoretical and Applied Sciences, eCampus University, 22060 Novedrate, Italy; 4 Centro di Ricerca in Psicologia Applicata (CePsi), eCampus University, 22060 Novedrate, Italy; 5 IUSS Cognitive Neuroscience (ICoN) Center, Scuola Universitaria Superiore IUSS, 27100 Pavia, Italy; anna.vedani@iusspavia.it (A.V.); gaia.locatelli@iusspavia.it (G.L.); 6 Cognitive Neuroscience Laboratory, Istituti Clinici Scientifici Maugeri IRCCS, 27100 Pavia, Italy; 7 Ph.D. Program in Neuroscience, School of Medicine and Surgery, University of Milano-Bicocca, 20900 Monza, Italy; b.visiello@campus.unimib.it; 8 Department of Psychology, University of Milano-Bicocca, 20126 Milan, Italy; leonor.romero1@unimib.it

**Keywords:** chronic primary pain, central sensitization, non-invasive brain stimulation, TMS, tDCS, chronic pain

## Abstract

**Highlights:**

**What are the main findings?**

- This meta-analysis highlights that non-invasive brain stimulation improves pain intensity, emotional distress, and functional disability in CPP.
- While NIBS benefits persist for up to one month after the treatment, the effectiveness declines as the duration of the patient’s illness increases.

**What are the implications of the main findings?**

- NIBS is effective in modulating the three core symptoms of CPP in the short and medium term.
- The findings emphasize the need for standardized treatment protocols, consistent follow-up, and targeted interventions.

**Abstract:**

Background/Objectives: Chronic primary pain (CPP) is a new diagnostic category including chronic pain conditions lacking clinical signs or a clear etiopathogenetic origin. These disorders may share a common neural mechanism known as central sensitization, where nociceptive neurons become hyper-responsive to standard or subthreshold pain stimuli, resulting in pain hyper-sensitivity. In this context, non-invasive brain stimulation (NIBS) appears to be a promising tool for improving CPP symptoms by targeting maladaptive brain activity and connectivity. To date, the effects of NIBS on CPP symptoms remain unexplored. To fill this gap, we conducted a meta-analysis, investigating the effect of NIBS in improving the three core symptoms of CPP, namely pain intensity, emotional distress, and functional disability. Methods: Following PRISMA guidelines, we screened four databases up to February 2025 for English-language, peer-reviewed randomized clinical trials that included CPP patients treated with NIBS and reported pre/post or follow-up scores on validated measures of at least one core symptom. Quality of life was examined as an additional outcome. Results: Fifty-four studies were included, with 1371 participants receiving real stimulation and 1103 sham. Findings highlighted that real stimulation improved CPP symptoms immediately after treatment and at one-month follow-up. Meta-regressions showed that longer CPP duration reduced short-term effects on emotional distress and diminished all outcomes at one-month follow-up. Conclusions: Further research is needed to establish standardized NIBS protocols for CPP management, to investigate the effectiveness at longer follow-up periods, and to test whether combining NIBS with other interventions enhances treatment effectiveness and durability.

## 1. Introduction

Given its widespread impact and complexity, chronic pain has become a global public health priority [1], prompting recent efforts, such as the World Health Organization’s ICD-11 revision, to refine its classification and better distinguish between primary and secondary chronic pain conditions [2]. The chronic primary pain (CPP) category encompasses all disorders in which pain itself is the primary cause of the condition, typically idiopathic pain conditions or conditions in which there are no clinical signs to justify the presence of symptoms. Among these are migraine, persistent idiopathic facial pain, and widespread pain syndrome, commonly known as fibromyalgia. CPP is opposed to chronic secondary pain, in which pain is at least initially viewed as a symptom secondary to an underlying disease [3].

According to the ICD-11, CPP includes three core symptoms: (i) persistent pain lasting longer than three months and affecting one or more anatomical regions, (ii) significant emotional distress, including anxiety, depressive symptoms, and the experience of negative emotions like anger and frustration, and/or (iii) functional disability, including the interference in daily activities or social functioning. Crucially, CPP can be diagnosed only when the symptoms cannot be sufficiently accounted for by any other condition.

The new categorization aims to shift the focus from disorder-specific symptoms to the shared underlying mechanisms driving these conditions. Although the etiopathogenesis of pain in CPP is not fully understood, a widely accepted theory suggests that it may involve central sensitization. Central sensitization is a mechanism in which nociceptive neurons exhibit increased responsiveness to standard or even subthreshold input, leading to pain hypersensitivity [4]. From this perspective, the central nervous system may alter, amplify, or modulate the perception of noxious stimuli. Consequently, pain perception no longer corresponds to the structural characteristics of harmful peripheral stimuli but is shaped by the altered neoplastic processes. This mechanism has been identified across various chronic pain conditions that are now included in the CPP category [5,6], thus reinforcing the hypothesis of a common neurophysiological substrate across chronic pain-specific disorders.

Neuroimaging studies provided converging evidence of widespread structural and functional anomalies in individuals with specific conditions now encompassed by CPP, compared with healthy participants. These findings underscore the central nervous system’s role in the experience, development, and maintenance of pain in the disorder [7,8,9,10,11,12,13,14,15]. Specifically, structural alterations involve the reduction in gray and white matter volume and cortical thickness in regions implicated in nociceptive processing and in the emotional-affective and regulatory components of the pain network, including the insula, amygdala, (hypo)thalamus, (para)hippocampus, anterior cingulate cortex, and precentral and inferior frontal gyri [10,12,16,17]. Anomalies in functional connectivity within a large-scale brain network have also been reported. These alterations involve the default mode, salience, and central executive networks, and extend to regions implicated in cognitive and affective processing (see [15,18] for reviews). Such complex and still not fully understood neural mechanisms highlight the multifaceted nature of CPP, which includes not only altered pain experience but also impairments in the emotional and cognitive domains [9,11,19,20,21,22].

When considering a chronic pain treatment strategy, pharmacological interventions are typically the first-line clinical choice [23]. Analgesics used across CPP-included disorders are centrally acting drugs, such as nonopioid analgesics, antidepressants with analgesic functions, gabapentinoids, or opioids [24,25,26,27,28,29,30]. However, pharmacological trials yielded unsatisfactory outcomes, with often limited pain relief and a high prevalence of side effects [31,32]. Therefore, an increasing demand for alternative or add-on multimodal strategies has emerged [33].

Over the past decades, non-invasive brain stimulation (NIBS) techniques gained considerable attention for modulating maladaptive brain activity and connectivity in various psychiatric and neurological conditions [34,35,36,37,38]. Transcranial magnetic stimulation (TMS) and transcranial electrical stimulation (tES) represent the most frequently used techniques. TMS delivers a strong, short magnetic pulse to the participant’s head, which induces neuronal firing by depolarizing the neuronal membrane suprathreshold [39]. TMS is typically delivered via repetitive pulses (rTMS) or patterned protocols (e.g., intermittent or continuous theta-burst stimulation, iTBS/cTBS) to induce long-lasting effects. Specifically, iTBS involves short bursts of high-frequency pulses administered at a slower, theta-range frequency (around 5 Hz), mimicking the brain’s natural rhythms [40].

TES includes neuromodulatory techniques in which weak, constant, or alternating currents are applied to the human brain through scalp electrodes. The most used tES techniques are transcranial direct current stimulation (tDCS) and transcranial alternating current stimulation (tACS). TDCS acts by delivering a constant current (typically 1–2 mA) through two electrodes, a positive (anode) and a negative (cathode) one [41]. The current is too weak to generate action potentials per se; instead, it induces small changes in membrane potential, thereby influencing the likelihood of neuronal spiking and, in turn, cortical excitability [42,43]. Traditionally, the parameters used to determine whether rTMS and tDCS protocols would produce inhibitory or excitatory effects are frequency for rTMS and polarity for tDCS. Low-frequency rTMS (≤1 Hz and continuous theta-burst stimulation) and cathodal tDCS are considered inhibitory protocols. In contrast, high-frequency rTMS (>5 Hz and intermittent theta-burst stimulation, iTBS) and anodal tDCS are considered excitatory [44,45]. In tACS, the current alternates at a specific frequency, producing a voltage that cyclically and gradually shifts between positive and negative polarity, thereby modulating physiologically relevant brain oscillations [46] that influence cortical neurons [47]. Although a detailed discussion of NIBS features and functioning is beyond the scope of the current work, it should be stressed that the outcomes of NIBS in terms of excitability or behavioral modulation are difficult to predict in advance. NIBS outcomes likely depend on the complex interplay among stimulation protocol parameters (intensity, duration, number of sessions), the brain regions stimulated, and their connections within the network, the state of the brain during the stimulation, and individual differences including anatomical, hormonal, and genetic features [48,49,50].

The rationale for using NIBS in CPP relies on NIBS’s potential to modulate cortical plasticity through synaptic strengthening (long-term potentiation) or weakening (long-term depression) processes [51,52] that outlast the stimulation time, thereby rebalancing the previously mentioned maladaptive activity and connectivity patterns [53,54,55,56] involved in pain processing and experience [57].

Recent reviews [58] and meta-analyses [59,60] on randomized clinical trials have discussed the application and quantitative effects of NIBS in specific chronic pain disorders included in CPP. For instance, Hou et al. (2016) [60] investigated the effects of sixteen rTMS and tDCS studies in which NIBS was applied as an add-on treatment to medications in patients with fibromyalgia. Overall, the results suggest that NIBS effectively reduced pain symptoms. Additionally, rTMS over the primary motor cortex (M1) alleviated fatigue, whereas rTMS over the dorsolateral prefrontal cortex (DLPFC) modulated depressive symptoms [60]. Feng et al. (2019) [59] included nine studies investigating the effects of NIBS on patients with migraines. Their results highlight that excitatory stimulation of M1 reduced pain intensity and headache attack frequency after the treatment [59]. Finally, Brighina et al. (2019) [58] discussed the use and impact of tES in patients with fibromyalgia. The authors reviewed 12 studies and suggested that delivering anodal tDCS over the left or bilateral M1 could reduce pain intensity but did not modulate performance in cognitive tasks involving executive functions and attention, or symptoms related to the affective domain, including anxiety, depressive symptoms, fatigue, and sleep disturbances. Conversely, the five studies that applied tDCS over the left or bilateral DLPFC did not report changes in pain perception. However, they showed a slight improvement in the previously cited cognitive and affective domains [58].

Taken together, previous data synthesis works suggested the effectiveness of NIBS in treating specific chronic pain conditions, such as fibromyalgia and migraine. However, they overlooked less common pain disorders now classified under the CPP category. According to the central sensitization hypothesis, these disorders may share common neural substrates and mechanisms and could therefore benefit from NIBS. Furthermore, previous quantitative analyses investigated only pain intensity or, in the case of migraine, attack frequency, neglecting emotional distress and/or the functional impact of the disease on patients’ lives, symptoms that are now included in the criteria required for CPP diagnosis. Finally, previous work has focused on pre- to post-treatment changes, whereas follow-up assessments have been considered less despite their importance in clarifying the durability of treatment [42,61].

Considering these points, the current work aims to qualitatively characterize and quantitatively measure the effectiveness of NIBS as a therapeutic strategy to improve the core symptoms of CPP, namely pain intensity, emotional distress, and functional disability. Effects on quality of life were also assessed in a subset of studies that included this measure. We considered the outcome measures recorded immediately after the treatment and, when possible, at follow-up. We further explored whether NIBS effects varied with specific stimulation parameters.

## 2. Materials and Methods

The systematic review and meta-analysis followed the Preferred Reporting Items for Systematic Reviews and Meta-Analyses (PRISMA) guidelines [62] (see the PRISMA Checklist in the Supplementary Materials).

Study quality assessment followed the Cochrane Collaboration’s Risk-of-Bias tool for RCT [63] (see Supplementary Materials—Section A for details).

### 2.1. Literature Search

Four databases, namely PubMed, Web of Science, Embase, and Scopus, were screened to identify peer-reviewed English-language original studies published up to February 2025 that investigated NIBS in patients with CPP. Keywords related to NIBS were combined with relevant primary pain labels identified using the MeSH Tree Structures in PubMed (see Supplementary Materials—Table S1). Papers were excluded when: (i) they were written in languages other than English; (ii) they were not original studies (e.g., case reports, reviews, meta-analyses, etc.); (iii) they included non-human samples or (iv) non-idiopathic pain pathologies; (v) did not use NIBS; or (vi) applied NIBS without a therapeutic purpose. Moreover, we excluded studies (vii) different from randomized clinical trials (RCT), (viii) not including a sham/placebo condition, and (ix) not including the necessary data to run the meta-analysis.

### 2.2. Records Screening and Data Extraction

We used the web and mobile systematic reviews manager, Rayyan web applications (https://rayyan.qcri.org/ accessed on 7 February 2025) [64] to conduct a blinded screening process. After removing duplicates, four researchers (A.T., A.Ved., G.L., and B.V.) independently screened the articles based on titles and abstracts, categorizing the items as “include”, “exclude”, or “maybe” according to the previously outlined criteria. The included articles were screened for the full text using the same blind procedure. When full texts were not available, we contacted the corresponding authors. Conflicting decisions during the title, abstract, and full-text screening phases were resolved through consensus. Possible discrepancies were solved with the agreement of all the authors. Then, B.V. and G.L. extracted data from the included articles using a structured form, which was checked for consistency and accuracy by other authors (A.T., A.Ver., A.Ved.).

The primary outcome measures reflected the three CPP core symptoms as outlined by the ICD-11, namely pain intensity, emotional distress, and functional disability before and after the treatment. Secondary outcome measures included follow-up data and participants’ reported quality of life. A detailed description of the measures included is provided in the Supplementary Materials, Section A.

### 2.3. Quantitative Analyses

We extracted relevant information for each article, comprising NIBS protocol features, the number of patients in the experimental and control groups, their demographic and clinical features, and the means and standard deviations for pre-, post-treatment, and follow-up outcome measures. We contacted the authors whenever data necessary for the analyses were missing or insufficiently reported. If the requested information could not be obtained from the authors and was available in graphical format, data were extracted using the free software WebPlotDigitizer (https://automeris.io/WebPlotDigitizer/ accessed on 5 May 2025).

For the different outcome measures, we computed the pre–post-treatment mean difference for the experimental and control groups by subtracting pre-treatment from post-treatment measures. Because higher scores reflect higher pain intensity, emotional distress, and functional disability, but lower quality of life, negative effect sizes indicated improvement in the former outcomes, and worsening in quality of life. We calculated the standard deviation of the pre–post-treatment difference according to the Cochrane Handbook for systematic reviews of intervention guidelines [65]:

$$SDchange=\sqrt{{SDpre}^{2}+{SDpost}^{2}-(2\times corr\times SDpre\times SDpost)}$$

where corr represented the correlation between pre- and post-measurement variances and was set at 0.5 following Follmann and colleagues [66]. However, we ran sensitivity analyses, assuming lower (0.25) and higher (0.75) correlations [67] to ensure this choice did not influence our results.

We computed the sampling variance and standardized mean difference (SMD) for each included study using the escalc function of the metafor package for R, version 3.4.3 [68]. The SMD function automatically corrects for positive bias due to small groups [68,69], computing Hedge’s g, which is used in the present work as an effect size measure.

Among the included studies, some provided sufficient information to calculate multiple effect sizes per outcome measure. For instance, regarding pain intensity, a few papers included more than one group receiving NIBS over different target regions [70,71]. Similarly, emotional distress measures often included separate effect sizes of anxiety and depressive symptoms [72,73,74]. Considering these effect sizes as statistically independent would violate the independence assumption of traditional meta-analyses and bias the statistical findings [75,76,77]. Therefore, to address this issue, we ran multilevel random-effects models using the rma.mv function in the metafor package [78], clustering the individual effect sizes at the study level. Then, in line with methodological guidelines [76], we compared the multi-level model with a reduced model (not including the three-level) using the anova function. We then applied the best-fitting model to analyze the data. When no differences emerged between the two models, we accepted the simpler one [76]. In this case, we employed a random-effects model using the rma function from the metafor package. We chose a random-effects model because it is suitable for addressing heterogeneity arising from sampling error and between-studies variance in effect sizes [79].

We provided several measures to display data heterogeneity [76]. We reported the variation due to the sampling error (Q statistic), the percentage of variation between studies not linked to the sampling error (I^2^ statistics) [80], and the prediction intervals (PIs) [81], an interesting measure that provides a range within which one can expect future studies’ effects to fall, based on the currently available data.

We planned to identify potential outliers and influential cases using the influence function inf implemented in the metafor package for the two-level analyses [68]. In the multi-level models, studies with studentized residuals higher than 2 were identified as outliers [82]. As recommended by previous authors [68], when we detected extreme values, we removed them and refitted the model to verify that their elimination did not impact the analysis results. Then, we ran meta-regression analyses to investigate the effects of potential categorical and continuous variables that could explain heterogeneity and the magnitude of extreme values. Potentially interesting moderators were defined a priori and included the specific stimulation technique (tDCS vs. rTMS), the target region (M1 vs. DLPFC), the protocol type (inhibitory vs. excitatory), the specific disorder, and the blinding (single vs. double). The number of sessions, TMS pulses, and illness duration were hypothesized as possible continuous predictors. However, the uneven distribution of study characteristics across the included papers prevented the inclusion of all hypothesized factors. Consequently, only moderators supported by sufficient studies were examined. As a rule of thumb, the Cochrane guidelines recommend conducting subgroup analyses and meta-regressions only when at least ten studies are available [83]. Given the exploratory nature of moderators in our heterogeneous sample of studies, we conducted subgroup analyses when at least six studies per group were available (for the number of effect sizes included in each analysis, see Tables S6–S17 column k in the Supplementary Materials).

Considering publication bias analyses, guidelines suggest avoiding them when between-study heterogeneity is high (I^2^ ≈ 75%), as results are unreliable [76,84]. When heterogeneity was lower, we used a modified version of the Egger regression test, as outlined by Pustejovsky and Rodgers (2019) [85] (see [76]). Indeed, for SMD effect sizes, the Egger regression test can inflate false-positive results due to the non-independence of standardized mean differences and standard errors.

## 3. Results

### 3.1. Studies Selection

**Figure 1:**
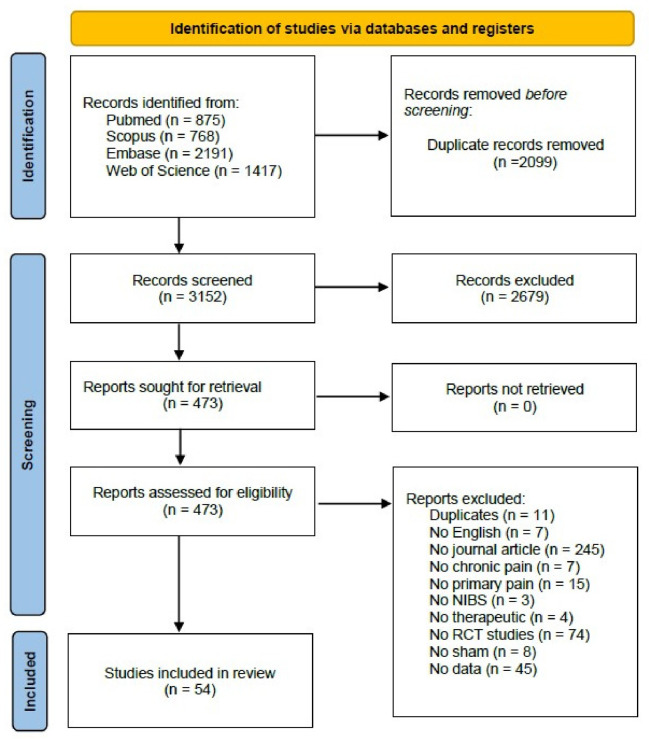
Flowchart of study selection.

The research intercepted 5251 records. We removed 2099 papers as duplicates and 2679 according to the exclusion criteria. We screened 473 full-text articles, and after double-blind checking, 54 studies were included in the meta-analysis. Figure 1 summarizes the selection procedure.

### 3.2. Studies Quality Assessment

**Figure 2:**
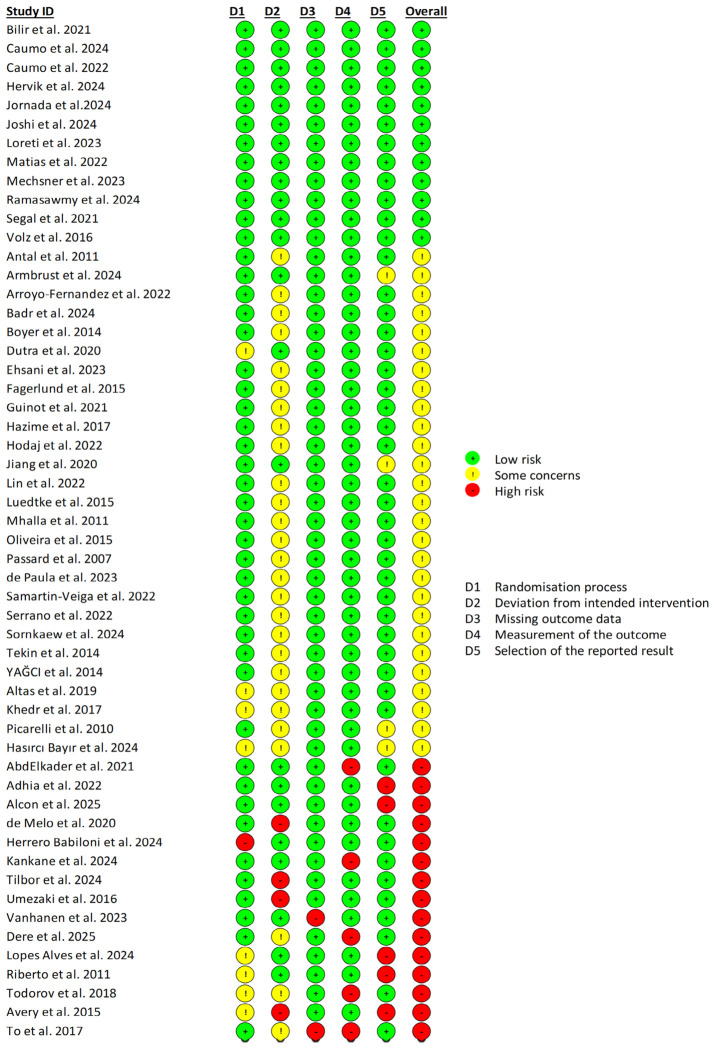
The graphical representation of risk of bias assessed for the 54 studies included in the meta-analysis [70,71,72,73,74,86,87,88,89,90,91,92,93,94,95,96,97,98,99,100,101,102,103,104,105,106,107,108,109,110,111,112,113,114,115,116,117,118,119,120,121,122,123,124,125,126,127,128,129,130,131,132,133,134].

We calculated the percentage of high-risk judgments to obtain a quality score for each study. Twelve studies (22.3%) were assessed as low risk, 15 (27.7%) were assessed as high risk, and the others were evaluated as having some concerns (50%). The most critical domain was deviation from the intended intervention, as many studies experienced dropouts and did not conduct intention-to-treat analyses. Since only randomized controlled trials were selected for the meta-analysis, the randomization process was among the domains with the lowest risk of bias. Nonetheless, some concerns emerged in specific studies, particularly related to the allocation sequence. In some cases, it was unclear when the allocation sequence was concealed [70,86,87,88], while in others, baseline differences between intervention groups were reported [89,90]. The domains of missing outcome data and the measurements of the outcome, both assessing the appropriateness and completeness of outcome data, were generally rated as low risk of bias, suggesting that researchers adequately and completely assessed the planned outcome measures and that outcomes were unlikely to be influenced by the raters’ knowledge of the intervention administered. The quality assessment results are reported in Figure 2.

### 3.3. Patients’ Demographic and Clinical Features

In total, 54 studies were included in the meta-analysis, involving 1371 patients with CPP assigned to stimulation groups and 1103 to control sham groups. Participants ranged in age from 21 to 64 years, with a mean age of 32.25 ± 13.54 years in the real stimulation group and 30.97 ± 81.59 years in the sham stimulation group. In most studies, the number of females was greater than that of males, in line with studies reporting a higher prevalence of women suffering from CPP [22,135]. Moreover, 23 studies included exclusively women.

The studies’ inclusion criteria required participants to be adults (over 18 years old) with a medical diagnosis of chronic pain, defined as pain lasting more than 3 months [136]. Exclusion criteria generally concerned contraindications to NIBS, neurological and psychiatric comorbidities, pregnancy and breastfeeding, a history of substance abuse, and a history of surgery or implanted devices.

The studies concerned several chronic pain conditions that are currently included in the CPP category. Specifically, 29 studies included patients with chronic widespread pain (i.e., fibromyalgia), 9 included chronic primary headache or orofacial pain (chronic migraine [70,91,92,93,94,95], temporomandibular disorder [96,97], burning mouth syndrome [98]), 10 included chronic primary musculoskeletal pain (chronic low back pain [73,99,100,101,102,103,104,105], phantom limb pain [106], 1 chronic neck pain [107]), 2 chronic primary visceral pain (irritable bowel syndrome [108], primary dysmenorrhea [89]), 2 complex regional pain syndrome [109,110], and one study reporting the diagnosis of persistent somatomorphic pain disorder, according to the ICD-10 DCR criteria [111]. As the ICD-11 classification of CPP was introduced only recently, none of the included studies enrolled participants with a formal CPP diagnosis. Consequently, the conditions included were retrospectively classified according to ICD-11 criteria by one of the authors (A.T.).

Table 1 and Table 2 summarize patient characteristics and diagnoses, separately for studies applying TMS (Table 1) and tES (Table 2).

### 3.4. NIBS Protocol Features: TMS Studies

Considering protocols applying TMS, 19 studies were included. RTMS was applied in all studies except two. In the paper by Joshi et al. [111], iTBS was used. Tilbor and colleagues [119] applied deep TMS (dTMS) using an H-coil to reach deeper, more widespread brain areas than traditional TMS [137].

A total of 17 studies chose an excitatory strategy, five targeting the left DLPFC [74,90,91,98,115] and seven targeting M1 (4 studies targeted the left M1 [113,116,117,118], 1 study targeted the contralateral M1 of the hand representation to the most painful face side [97], 1 study targeted the M1 representing the dominant hand [114] and 1 study did not specify the M1 side [109]). Moreover, three studies included two independent groups undergoing real stimulation delivered over the left M1 or the left DLPFC, respectively [70,87,111]. One study targeted the somatosensory cortex (S2) [110], and one simultaneously targeted the medial prefrontal cortex (mPFC) and the anterior cingulate cortex (ACC), using the H-coil [119]. Stimulation frequencies ranged from 5 to 20 Hz, with durations of 5 to 37.5 min, and the total number of pulses per session ranged from 600 to 3000. The number of sessions ranged from 1 to 20. The stimulation intensity ranged from 70% [70,138] to 120% [90,112] of the resting motor threshold (rMT), which is the minimum intensity required to evoke a motor response in the target muscle greater than 50 μV in at least 50% of trials [139].

Only three studies applied an inhibitory protocol [112,115,120]. One study included two independent groups undergoing inhibitory (1 Hz) protocols on the left DLPFC or excitatory (10 Hz) protocols on the right DLPFC [115].

No major adverse reactions were reported. Mild and transient side effects, such as headache, tinnitus, dizziness, and neck pain, were reported in 19 studies (~35%), with no significant difference between the real stimulation and sham stimulation groups. Sham stimulation typically consisted of using a sham/placebo coil to mimic the stimulation noise, different inclinations of the coil, or a reduced stimulator output intensity. TMS protocol features are summarized in Table 3 and in the Supplementary Materials (Table S3).

### 3.5. NIBS Protocol Features: tES Studies

Of the 35 studies applying tDCS, 28 employed anodal stimulation, 2 applied the cathodal protocol [93,101], and 7 studies used a bihemispheric protocol [71,122,123,126,128,133,134], of which three studies applied bihemispheric and excitatory protocols in two different groups [71,128,134]. In line with the previous literature, we refer to ‘anodal’ tDCS for studies positioning the anode over the relevant region stimulated to modulate symptoms (and therefore using an excitatory strategy), to ‘cathodal’ tDCS when the cathode was positioned over the target area (thus applying an inhibitory strategy), or bi-hemispheric when the stimulation strategy consisted of the synchronous and polarity-opposite stimulation of two brain regions. Considering the target region, 12 studies targeted the left M1 [72,73,86,88,92,95,121,124,127,129,130,132], 6 the M1 contralateral to the painful area [96,99,100,104,106,108], and 4 the M1 corresponding to the hand-dominant hemisphere [102,107,125,131]. Six studies targeted the left DLPFC [71,89,94,102,122,123], and four targeted the DLPFC bilaterally, delivering anodal stimulation over the left and cathodal over the right (F3 with F4 cathode per EEG 10-20 IS) [122,123,133,134]. Two studies used two montages: in the first, the target was the DLPFC bilaterally, and in the second, the target was the left M1 [126,128]. One study compared three montages: left M1, left DLPFC, and occipital cortex with a multielectrode montage (six electrodes placed in F3, FC1, F8, FC5, C5, and P3) [71]. Moreover, two of the included studies used an extracephalic montage applying the cathode on the contralateral arm [86,134]. In particular, To and colleagues [134] applied the extracephalic montage to stimulate the occipital nerve dermatomes (left anode, right cathode) with an excitatory protocol, and also a bihemispheric montage targeting the DLPFC; Khedr and colleagues [86] instead, stimulated the left M1, placing the anode in C3 and the reference electrode (i.e., cathode) on the contralateral arm.

TDCS intensity varied from 1 to 2 mA, with electrode sizes ranging from 25 cm^2^ to 35 cm^2^, and current density averaging around 0.10 (range: 0.025–0.5). The number of sessions ranged from 1 to 20, with session durations of 15 to 22 min. One study applied tACS, targeting the left M1 with monophasic square waves [127], and one study [105] administered the High-Definition Transcranial Infraslow Pink-Noise Stimulation (HD-tIPNS), which uses low-frequency pink noise stimulation to specifically modulate the infraslow electrical activity (0.0–0.1 Hz) in the brain [105,140,141]. Finally, five studies of the included studies implemented a home-based protocol [123,126,132].

In line with safety guidelines [142], no major adverse reactions were reported. In total, 26 (~75%) of the studies reported mild, transient side effects, including headache, neck pain, tingling, and skin redness. Sham stimulations were typically delivered using the same devices and identical electrode placement. In sham, the current was turned off after a few seconds of stimulation, typically 30 s [143]. TES protocol features are summarized in Table 4 and in the Supplementary Materials (Table S4).

Therapeutic strategies from TMS and tES studies are discussed in the Supplementary Materials—Section C.

### 3.6. Quantitative Results

For the sake of clarity, results from the primary outcome measures, namely pain intensity, emotional distress, and functional disability before and after the treatment, are presented in the following paragraphs. Secondary outcome measures, including the effect of NIBS on the three core CPP symptoms at one-month follow-up and quality of life, are presented in Supplementary Analysis—Section C.

#### 3.6.1. Primary Outcome Measure: Short-Term Effects of NIBS on Pain Intensity

**Figure 3:**
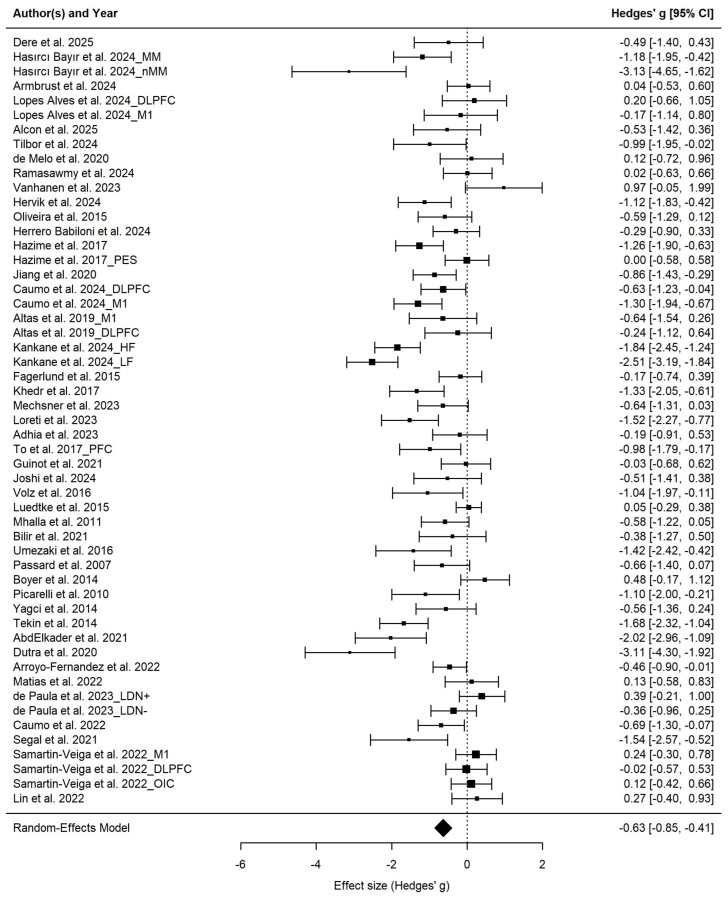
Forest plot of the short-term effect size of NIBS on pain intensity. Squares represent individual study effect sizes, with sizes proportional to study weights; solid horizontal lines indicate the corresponding 95% CIs. The vertical dashed line indicates no effect. The diamond represents the pooled three-level random-effects estimate and its 95% CI. Negative values indicate greater pain reduction following real NIBS compared with sham/control stimulation [2,3,5,7,13,14,22,25,30,31,38,39,42,48,53,65,67,68,72,73,86,90,91,93,101,103,105,107,114,115,117,132,138,145,146,147,148,149,150,151,152,153,154,155].

Forty-four studies were included, containing fifty-three effect sizes. The three-level model provided a significantly better fit than the reduced one (see Table S5 in the Supplementary Materials for details). The meta-analysis results are summarized in the forest plot (Figure 3). The random effects model showed a significant effect *g* = −0.63, 95% confidence interval (CI) [−0.85, −0.41], t = −5.67, *p* < 0.001, suggesting that real stimulation has a moderate to large impact on reducing participants’ pain experienced after the treatment. The estimated variance components were τ^2^_Level 3_ = 0.317 and τ^2^_Level 2_ = 0.106, with I^2^_Level 3_ = 58.3% of the total variation attributed to the between-cluster level, and I^2^_Level 2_ = 19.5% to within-cluster heterogeneity. Three datapoints were identified as influential cases: Dutra et al. 2020 [89], Hasirci Bayr et al. [94] for the non-Menstrual Migraine group receiving tDCS on the left DLPFC, and the group of Kankane et al. [115] receiving low-frequency rTMS. The effect sizes removal, however, did not change the overall significance: g = −0.55, 95% CI [−0.74, −0.35], t = −5.71, *p* < 0.001.

The included moderators and covariates did not highlight significant patterns (see Tables S6 and S7 for details). Publication bias was not explored due to the substantial total heterogeneity (I^2^ = 77.75%).

#### 3.6.2. Primary Outcome Measures: Short-Term Effects of NIBS on Emotional Distress

**Figure 4:**
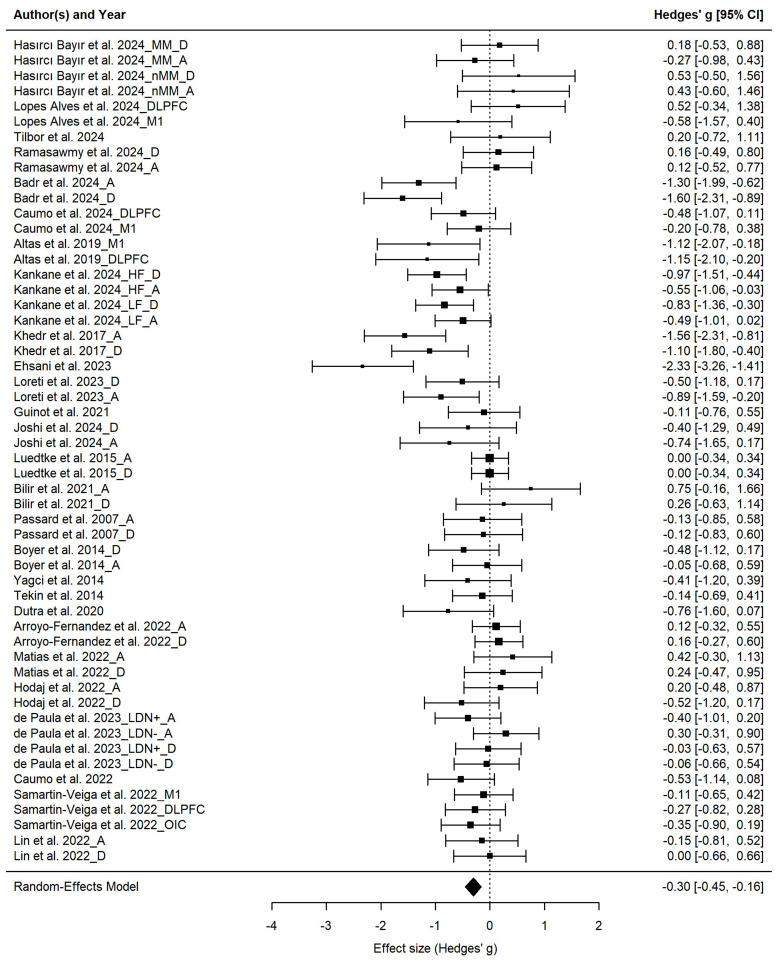
Forest plot of the short-term effect size of NIBS on emotional distress. Squares represent individual study effect sizes, with sizes proportional to study weights; solid horizontal lines indicate the corresponding 95% CIs. The vertical dashed line indicates no effect. The diamond represents the pooled random effects estimate and its 95% CI. Negative values indicate a greater reduction in emotional distress following real NIBS compared with sham/control stimulation [7,14,17,22,25,30,31,39,48,49,65,67,76,90,91,93,101,103,105,107,114,138,146,147,149,150,155].

**Figure 5:**
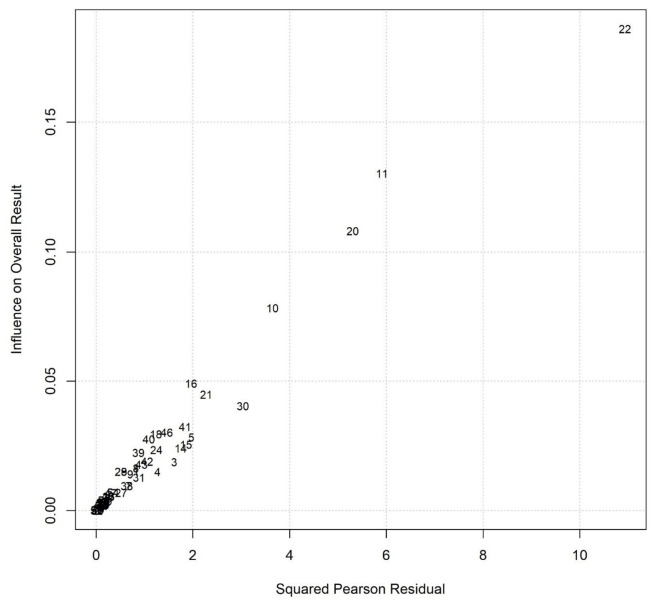
Baujat plot of study contributions to heterogeneity for pre–post emotional distress.

Twenty-seven studies were included, containing forty-four effect sizes. The chosen model was the reduced one (see Table S5). The meta-analysis results are summarized in the forest plot (Figure 4). The random effects model was significant, *g* = −0.30, 95% CI [−0.45, −0.16], t = −4.30, *p* < 0.001. This result suggests that real stimulation may have a small but significant impact on reducing participants’ emotional distress experienced after the real stimulation. The meta-analysis also revealed moderate heterogeneity between studies, Q _(53)_ = 131.54, *p* < 0.001, τ^2^ = 0.15 (SE = 0.05), and I^2^ = 59.71% [48.29; 78.73]. PIs [−1.10, 0.49] crossed zero, thus indicating that null or ‘negative’ treatment effects cannot be ruled out for future studies. The Baujat plot inspection (Figure 5) suggested that in one study [97], the anxiety measure contributed to the heterogeneity of the statistical analysis, and the influence analysis confirmed this datapoint as an influential case. The effect size removal, however, did not alter the overall significance: g = −0.28, 95% CI [−0.41, −0.14], t = −4.15, *p* < 0.001.

Considering the publication bias, the modified Egger test showed no asymmetry b = −0.20, 95% CI [−1.50, 1.10], t = −0.19, *p* = 0.853.

Subgroup analysis showed a trend toward significance for NIBS type (*p* = 0.062), likely due to larger effect sizes (and smaller number of included studies) of rTMS studies compared to tDCS ones (see Table S8). The meta-regression analysis indicated a significant effect (*p* = 0.007) for the illness duration predictor, indicating that for each additional year of illness, the impact of real stimulation in reducing emotional distress becomes less effective. Moreover, the opposite trend was found for the number of sessions (*p* = 0.059) (see Table S9), where increasing the number of stimulation sessions augmented the effectiveness of real stimulation.

#### 3.6.3. Primary Outcome Measures: Short-Term Effects of NIBS on Functional Disability

**Figure 6:**
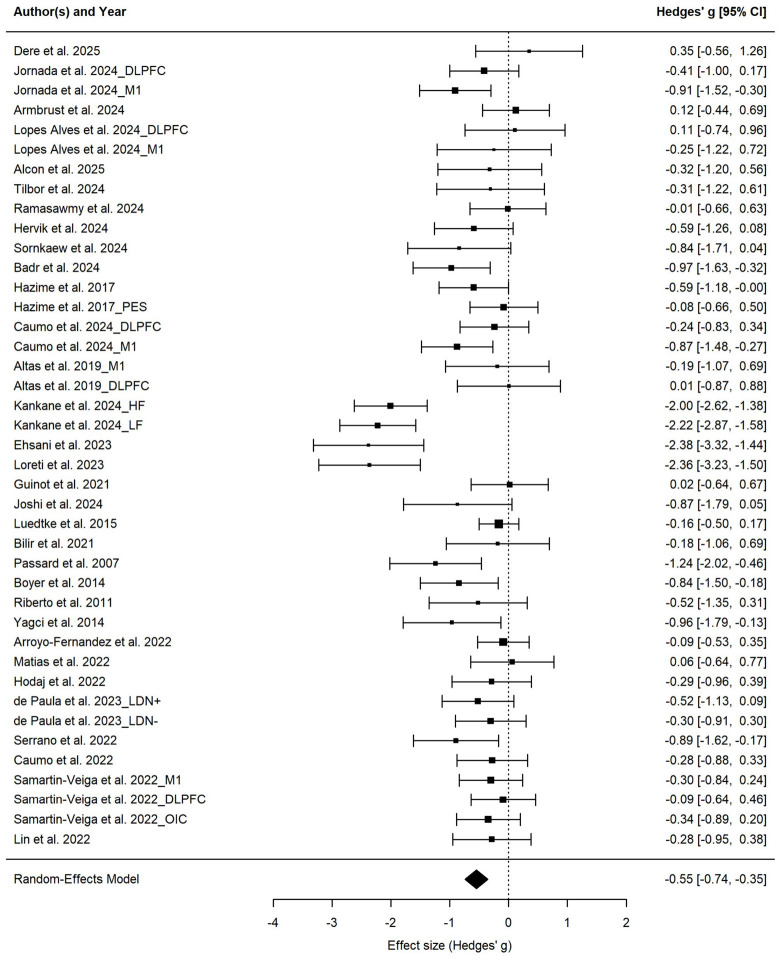
Forest plot of the short-term effect size of NIBS on functional disability. Squares represent individual study effect sizes, with sizes proportional to study weights; solid horizontal lines indicate the corresponding 95% CIs. The vertical dashed line indicates no effect. The diamond represents the pooled random effects estimate and its 95% CI. Negative values indicate a greater reduction in functional disability following real NIBS compared with sham/control stimulation [5,7,13,14,17,22,25,30,31,39,42,49,65,68,73,76,89,90,91,103,104,105,107,114,138,146,147,150,155,156,157,158].

**Figure 7:**
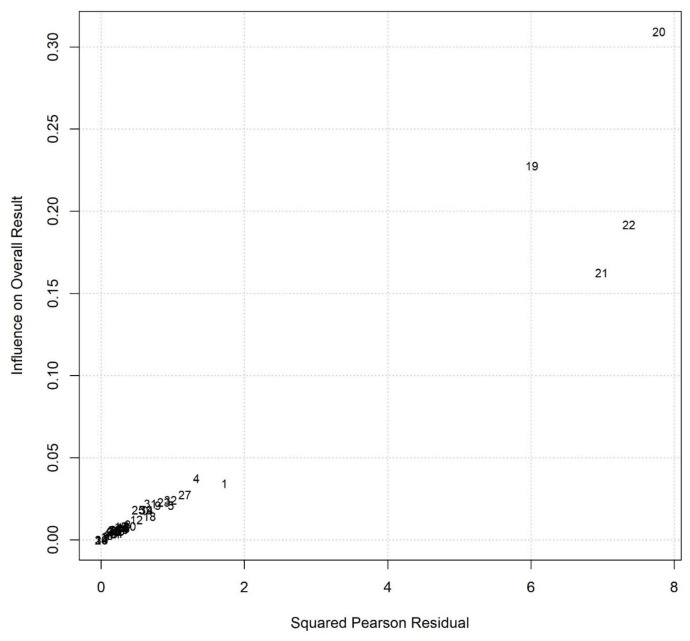
Baujat plot of study contributions to heterogeneity for pre–post functional disability.

Thirty-two studies were included, containing forty-one effect sizes. The chosen model was the reduced one (see Table S5). The meta-analysis results are summarized in the forest plot (Figure 6). The random effects model was significant g = −0.55, 95% CI [−0.74, −0.35], t = −5.67, *p* < 0.001. This result suggests that real stimulation has a small but significant impact on reducing participants perceived functional disability after the stimulation. The meta-analysis also revealed moderate to substantial heterogeneity between studies, Q _(40)_ = 131.44, *p* < 0.001, τ^2^ = 0.25 (SE = 0.09), and I^2^ = 69.57% [57.82; 83.79]. PIs [−1.58, 0.49]. Baujat plot inspection (Figure 7) suggested that Kankane et al. [115] LF and HF, Ehsani et al. [101], and Loreti et al. [129] as potential outliers. The influence analysis confirmed that Kankane et al. [115] LF and HF were influential cases. The effect size removal, however, did not change the overall significance: g = −0.45, 95% CI [−0.61, −0.29], t = −5.69, *p* < 0.001.

The included moderators and covariates did not highlight significant patterns (Tables S10 and S11). Considering the publication bias, the modified Egger test using Pustejovsky-Rodgers’ method was not statistically significant (b = −1.10, 95% CI [−3.07, 0.87], t = −0.55, *p* = 0.587), suggesting no evidence of publication bias.

## 4. Discussion

The CPP diagnosis has been included in the latest ICD revision (ICD-11) [2], based on the central sensitization hypothesis [4]. This theory posits that the central nervous system plays a crucial role in modulating pain processing and experience, thereby contributing to hypersensitivity. Emerging evidence supports this theory, showing common neural underpinnings across chronic pain conditions now included in the CPP [5,6]. By promoting plastic changes, NIBS has the potential to modulate maladaptive brain activity and connectivity, as evidenced by its capacity to modulate cortical plasticity through synaptic strengthening or weakening processes [51,52,144] that persist beyond the stimulation period, rebalancing the maladaptive activity and connectivity patterns involved in pain processing and experience [53,54,55,56,57].

Previous meta-analyses and reviews have explored the effectiveness of NIBS in reducing pain in specific disorders now included in CPP [58,59,60] or in chronic pain conditions regardless of their classification as primary or secondary conditions [145,159]. However, to our knowledge, no previous study has investigated the effects of NIBS on CPP as a unified category. To address this gap, we systematically reviewed and quantitatively analyzed the impact of NIBS on core CPP symptoms in the short- and medium-term. We included 54 sham-controlled RCTs, comprising 1371 participants who received real stimulation and 1103 who received sham stimulation. It is important to note that the available evidence is unevenly distributed across CPP subtypes, with chronic widespread pain accounting for the largest proportion of studies. Although Chronic Primary Musculoskeletal Pain and Chronic Primary Headache or Orofacial Pain are also relatively well represented, other CPP conditions have received only limited empirical attention.

As primary endpoints, we focused our analysis on the three core symptoms of CPP diagnosis, namely pain intensity, emotional distress, and functional disability [3], before and after intervention. Secondary outcomes included the pre–post effect of NIBS on quality of life in the short term and the three core CPP symptoms at one-month follow-up.

NIBS short-term effects, observed immediately after treatment, indicated stimulation-induced effects on all core symptoms. Results on pain intensity align with previous literature that reported pain intensity modulation after NIBS interventions in migraine [59], as an add-on therapy to drugs in fibromyalgia [60]. Even the effect on emotional distress, or more specifically, anxiety and depressive symptoms, is in line with previous evidence that suggested improvements in mood and anxiety scores [58,60]. Interestingly, previous studies suggested that the effects on pain severity and affective symptoms may depend on the targeted brain region. Specifically, Hou et al. [60] and Brighina et al. [58] reported that stimulating M1 was more effective in reducing pain intensity and fatigue, while targeting the left DLPFC improved depressive and anxiety symptoms [58,60]. Our findings did not indicate an effect of the target region on pain intensity or emotional distress scores. However, it should be noted that our dataset was unbalanced in this regard, as it included more studies targeting the left M1 than the left DLPFC, thereby preventing differences between the stimulated target regions from emerging. Real stimulation also showed a moderate, significant effect in improving functional disability scores. Interestingly, previous meta-analyses and reviews evaluating the effects of NIBS on this outcome measure have reported heterogeneous results, with some studies not reporting significant improvements [124,140] or not exploring the topic at all [58,59]. Interestingly, the effects on pain intensity and functional disability remained largely unchanged when only studies rated as at low risk of bias (N = 12) were considered, whereas the effect on emotional distress did not persist, suggesting a lower degree of robustness for this outcome.

Regarding the quality of life, our findings did not reveal any significant difference between real stimulation and sham. This finding is not unexpected, given the multidimensional nature of the construct [160,161] and the likelihood that improvements in quality of life occur indirectly through changes in core symptoms rather than as a direct consequence of stimulation. In addition, the limited number of studies and the heterogeneity of assessment measures may have further hindered the detection of significant effects.

Crucially, in the subset of studies reporting follow-up data (n = 19), the effects of real stimulation remained significant one month after the end of treatment across the three core symptom measures, ranging from small (emotional distress scores) to large (pain intensity and functional disability evaluations) effects. Previous meta-analyses typically did not examine the effects of NIBS on follow-up measures [59,60]. To our knowledge, only O’Connell et al. [145] reported that tDCS reduced chronic pain at follow-up measures between one and six weeks post-treatment, whereas no clear evidence emerged for rTMS. Current findings suggest that NIBS can induce short- and medium-term changes, consistent with the idea that metaplastic changes are induced by stimulation [162]. The exact mechanisms behind these improvements are not fully understood. The papers included in our work typically targeted M1 and the left DLPFC, which are extensively connected to cortical and subcortical brain structures [163,164] that are involved in multiple functions, from the regulation of cognitive and affective processes to the release of neurotransmitter systems that can ultimately affect the processing and experience of pain [146,165]. Therefore, the stimulation produces effects at the local and distal levels, inducing large-scale network modifications based on anatomical and functional connectivity [157,166,167,168,169,170,171]. For instance, previous studies have suggested that neurotransmitters (e.g., glutamate, GABA, and serotonin) [147,172,173] and endogenous opioids are released following M1 stimulation [146,148,158,174]. The stimulation of DLPFC, on the other hand, is known to modulate the activity of the anterior cingulate cortex [156,175], which has been associated with emotional and pain processing [176]. However, because follow-up assessments were inconsistently reported across the included studies, it is not possible to determine whether sustained effects are specific to the stimulation paradigms used or can be generalized. As such, this issue remains unresolved and requires further investigation. Moreover, future studies should clarify the pathological and healing neural mechanisms underlying CPP and determine whether one or more regions are optimal candidates for effective, sustained intervention.

Considering the categorical moderators, our data highlight differences between the choice of rTMS and tDCS protocols only in functional disability (and a trend in emotional distress), with larger effect sizes for rTMS. This is partially consistent with the most recently updated expert panels’ guidelines for the clinical use of NIBS in chronic pain [37,38], which recommended Level B evidence (‘probable efficacy’) for tDCS and Level A evidence (‘definite efficacy’) for rTMS analgesic effects on neuropathic pain. However, it should be noted that the studies included in our analysis were unbalanced between the two NIBS techniques, with approximately twice as many applying tDCS. No differences emerged between improvements in anxiety and depressive symptoms, suggesting that the improvements were not driven by one of the two measures. The similar response of anxiety and depressive symptoms is not surprising since they are highly correlated [149,177] and benefit similarly from NIBS intervention [36,178,179], possibly sharing some neural underpinnings [180].

We also explored the impact of continuous predictors, namely the number of sessions and illness duration. Illness duration was included by a subset of studies (N = 35), and it effectively modulated short-term NIBS effects on emotional scores: specifically, a reduction in mood and anxiety improvement was observed with real stimulation among patients with longer illness duration, suggesting that longer illness duration predicted reduced benefits from stimulation. Crucially, illness duration significantly predicted the effectiveness of stimulation on the three core symptoms at the one-month follow-up, with longer illness duration reducing the improvements induced by the stimulation. This finding is in line with previous evidence in which longer duration of (untreated) illness negatively affects treatment outcomes across several disorders, including schizophrenia [150,151], eating disorders [181], and obsessive-compulsive disorders [182]. Such an effect has also been reported in studies including CPP participants, specifically chronic low back pain [8] and fibromyalgia [21]. The link between longer illness duration and more negative treatment outcomes can be attributed to neuroplastic maladaptive changes [183] that may be more resistant to treatment and, in the current analysis, to NIBS. Of course, maladaptive neuroplastic changes are not the only factor explaining negative treatment outcomes; indeed, other mechanisms, including dysfunctional coping strategies such as catastrophizing thinking, rumination, helplessness feelings [152,184], cognitive impairment, such as attentional deficits, reduced working memory, and increased hypervigilance or fear [153,185,186,187], may take place in chronic pain disorders [154,184,188,189]. Observational studies showed that the observed avoidance behaviors or catastrophizing thinking, frequently reported in chronic pain patients, may exacerbate the experience of pain, the impact of the disease on individuals’ lives, and the maintenance of negative emotions and depression symptoms [155,190,191]. In line with this evidence, multimodal interventions are required to consider the disorder’s multifaceted nature [36,192], exploring the hypothesis that the effects of NIBS may be maximized by combining stimulation with behavioral, cognitive, or pharmacological interventions. For instance, cognitive/behavioral interventions may promote plastic changes at the brain level, addressing maladaptive coping strategies or dysfunctional processes through, for instance, psychotherapy interventions, such as cognitive-behavioral approaches, including acceptance and commitment therapy (NICE guidelines, 2021) [193], and such psychological changes can be further reinforced and sustained through NIBS-induced plasticity [194]. Consistent with the growing interest in multimodal treatment approaches, the studies included in our meta-analysis adopted a variety of treatment strategies. Following the framework proposed by Razza et al. [35], interventions were classified as monotherapy (when NIBS is the only treatment applied), add-on (when stimulation is added to the ongoing individual therapy, not manipulated within the study), or augmentative (when NIBS is combined with other interventions) (see the therapeutic strategy paragraph in the Supplementary Materials for a more detailed description). Notably, augmentative protocols encompassed a broad range of concomitant interventions, including physical, psychological, and pharmacological approaches. Given this variability, the treatment strategy was not examined statistically. Nevertheless, differences in this variable may have contributed to the heterogeneity observed across studies.

### Study Limitations

The current work presents several limitations. First, substantial heterogeneity emerged across the studies included at both the qualitative and quantitative levels. This variability likely reflects differences in several clinical and methodological features, including the CPP populations investigated, the treatment strategies adopted (i.e., monotherapy, add-on, and augmentative approaches), the instruments used to assess the different outcomes, and the stimulation protocols’ features. The latter variable varied considerably not only with the specific technique applied, but also with the targeted regions, the number and frequency of stimulation sessions, and stimulation and sham parameters. Regarding sham manipulation, participants’ blinding was not systematically assessed across studies, raising concerns about potential placebo effects resulting from differences in the credibility of sham procedures [195,196]. Therefore, while the pooled analyses support an overall beneficial effect of NIBS, the substantial between-study heterogeneity suggests that treatment effects may not be constant (or present) across clinical contexts, populations, and intervention protocols, warranting cautious interpretation of the pooled estimates.

A second limitation arises from the risk-of-bias assessment. As previously highlighted, only 12 of 54 studies (22.3%) were rated as low risk, suggesting the need for clearer operational guidelines for delivering NIBS in CPP disorders.

A third limitation arises from the CPP-specific distribution of diagnoses across studies. As previously highlighted, most studies referred to chronic widespread pain, followed by chronic musculoskeletal and chronic headache/orofacial pain. This uneven distribution limits the generalizability of the findings across the broader spectrum of CPP disorders. Future studies are needed to clarify whether NIBS can effectively improve symptoms across CPP diseases.

Fourth, anxiety and depressive symptoms have been analyzed as emotional distress measures. This was done since most studies included these questionnaires; however, it is essential to note that they represent only some possible manifestations of emotional distress.

Finally, the review was neither registered nor a protocol recorded. However, a priori decisions considering outcome measures and possible predictors are reported in the quantitative analysis paragraph.

## 5. Conclusions

In the current work, we systematically reviewed and quantitatively analyzed the effect of NIBS on CPP core symptoms, namely pain intensity, emotional distress, and functional disability. Overall, our findings suggest that NIBS can effectively reduce the core symptoms of CPP in the short and medium term. Future research should investigate the sustained effects of NIBS at longer intervals and explore combining NIBS treatments with behavioral, psychological, or pharmacological interventions. More studies are required to clarify the pathological and healing mechanisms of CPP to develop effective, evidence-based, and sustained interventions.

## Figures and Tables

**Table 1:** Participants’ demographic and clinical characteristics in the included TMS studies.

Authors, Year (Ref)	N Patients	Age	Sex	Education	Illness Duration (Years)	N Patients	Age	Sex	Education	Illness Duration (Years)	Diagnosis (Criteria)
**AbdElkader et al.** **2021 [91]**	16	34.19 ± 9.738	F: 13, M: 3	n.r.	1.83 ± 3	11	33.91 ± 8.526	F: 9, M: 2	n.r.	2.83 ± 0.71	Chronic tension-type headache and chronic migraine (IHS criteria-2004)
**Altas et al.** **2019 [87]**	M1: 10; DLPFC: 10	M1: 46.3 ± 9.01; DLPFC: 47.9 ± 7.89	F	n.r.	M1: 3 ± 1.83; DLPFC: 4.2 ± 1.14	10	48.2 ± 9.38	F	n.r.	3.6 ± 1.43	Fibromyalgia (ACR 2010)
**Avery et al.** **2015 [90]**	7	54.86 ± 7.65	F	n.r.	11 ± 4.26	11	52.09 ± 10.02	F	n.r.	15.64 ± 6.93	Fibromyalgia (ACR 1990)
**Badr et al.** **2024 [112]**	20	31.9 ±7.49	F: 11, M: 9	Illiterate: 8 (40%); Educated: 12 (60%)	8.30 ±3.17	20	34.55 ±8.32	F: 12, M:8	Illiterate: 7(35%); Educated: 13(65%)	6.60 ± 3.33	Fibromyalgia (ACR 2010)
**Bilir et al.** **2021 [74]**	10	46.70 ± 9.06	F	Primary school: 9 (90%); High school 1 (10%); University: 0	5 ± [2–5.3]	10	43.80 ± 9.37	F	Primary school: 6 (60%); High school 3 (30%); University 1 (10%)	5.5 ± [4–5.5]	Fibromyalgia (diagnostic criteria 2016)
**Boyer et al.** **2014 [113]**	19	49.1 ± 10.6	F	Secondary school: 9 (47.4%)	3.7 ± 4.5	19	47.7 ± 10.4	F: 18, M: 1	Secondary school: 13 (68.4%)	3.6 ± 3.8	Fibromyalgia (ACR 2010)
**Guinot et al.** **2021 [114]**	17	46.5 ± 10.4	F	9 C, 9 HS	11.2 ± 10.9	19	42.8 ± 8.8	F: 15, M: 4	12 C, 7 HS	9.2 ± 9.6	Fibromyalgia (ACR 2010)
**Herrero Babiloni et al.** **2024 [97]**	41	26.63 ± 8.57	F	26 (64.4%)	6.59 ± 9.83	n.a. ^a^	n.a. ^a^	n.a. ^a^	n.a. ^a^	n.a. ^a^	Temporomandibular disorder (diagnosis confirmed by a specialist)
**Joshi et al.** **2024 [111]**	11	45.73 ± 14.29	F: 9, M: 2	Not at all: 2; Primary: 2; Secondary: 5; Graduation: 2	9.82 ± 4.51	9	40.00 ± 15.26	F: 7, M: 2	Not at all: 2; Primary: 3; Secondary: 1; Graduation: 3	6.89 ± 3.44	Persistent somatomorphic pain disorder (ICD-10 DCR criteria)
**Kankane et al.** **2024 [115]**	HF-DLPFC TMS: 30; LF-DLPFC TMS: 30	HF-DLPFC TMS: 43.23 ± 14.71; LF-DLPFC TMS: 38.13 ± 7.33	n.r.	n.r.	HF-DLPFC TMS: 1.23 ± 0.62; LF-DLPFC TMS: 1.11 ± 0.52	sham DLPFC TMS: 30	42.77 ± 10.43	n.r.	n.r.	1.09 ± 0.69	Fibromyalgia (ACR 2010)
**Mhalla et al.** **2011 [116]**	20	51.8 ± 11.6	F	n.r.	13 ± 12.9	20	49.6 ± 10	F	n.r.	14.1 ± 11.9	Fibromyalgia (ACR 1990)
**Passard et al.** **2007 [117]**	15	52.6 ± 7.9	F: 14, M: 1	n.r.	8.1 ± 7.9	15	55.3 ± 8.9	F	n.r.	10.9 ± 8.6	Fibromyalgia (ACR 1990)
**Picarelli et al.** **2010 [109]**	11	43.5 ± 12.1	F: 7, M: 5	n.r.	6.9 ± 2.9	11	40.6 ± 9.9	F: 7, M: 4	n.r.	6.6 ± 2.7	Complex regional pain syndrome (CRPS) Type I (IASP)1
**Tekin et al.** **2014 [118]**	27	42.4 ± 7.63	F: 24, M: 3	8.40 ± 2.85	10.81 ± 6.31	24	46.5 ± 8.36	F: 23, M: 1	8.13 ± 2.42	13.33 ± 6.65	Fibromyalgia (ACR)
**Tilbor et al.** **2024 [119]**	8	51.7 ±12.8	F: 7, M: 1	15.6 ±1.9	n.r.	11	46.2 ±13.5	F: 9, M: 2	15.7 ±3.2	n.r.	Fibromyalgia (ACR 2011 Criteria)
**Todorov et al.** **2020 [70]**	M1: 38, lDLPFC: 37	M1: 40.2 ± 11.05; lDLPFC: 38.7 ± 11.05	M1: F: 31, M:7; lDLPFC: F:30, M: 7	M1: secondary: 21, university:17; lDLPFC: secondary:20, university: 17	M1: 19.3 ± 8.75; lDLPFC: 19.2 ± 8.42	sham TMS: 28; Topiramato:34	sham TMS: 36.9 ± 10.28; Topiramato: 39.6 ± 9.52	sham TMS: F: 22, M:6; Topiramato: F:25, M: 9	sham TMS: secondary: 16, university:12; Topiramato: secondary:19, university: 15	sham TMS: 18.5 ± 9.81; Topiramato: 20.5 ± 10.73	Chronic migraine (IHS criteria—2013)
**Umezaki et al.** **2016 [98]**	12	63.36 ± 10.78	F: 92.9%, M: 7.1%	n.r.	5.1 ± 2.7	8	64.42 ± 8.35	F: 91.7%, M: 8.3%	n.r.	5 ± 4.6	Burning mouth syndrome (BMS) (IHS criteria—2013)
**Vanhanen et al.** **2023 [110]**	21	46 ± 12	F: 18, M: 3	n.r.	2.2 (0.7–16)	n.a ^a^	7	n.a. ^a^	n.a. ^a^	n.a. ^a^	Complex regional pain syndrome criteria
**Yagci et al.** **2014 [120]**	12	45.25 ± 9.33	F	n.r.	4.42 ± 2.43	13	43 ± 7.63	F	n.r.	4.58 ± 2.54	Fibromyalgia (ACR 1990)

Notes: ^a^ = Mean divided by condition not available; ACR = American College of Rheumatology; C = College; DCR = Diagnostic Criteria for Research; DLPFC = dorsolateral prefrontal cortex; F = female; HF = high-frequency; HS = High school; IASP = International Association for the Study of Pain; ICD-10 = International Classification of Diseases, 10th Revision; IHS = International Headache Society; lDLPFC = left dorsolateral prefrontal cortex; LF = low-frequency; M = male; M1 = primary motor cortex; N = number; n.a. = not applicable; n.r. = not reported.

**Table 2:** Participants’ demographic and clinical characteristics in the included tES studies.

Authors, Year (Ref)	N Patients	Age	Sex	Education	Illness Duration (Years)	N Patients	Age	Sex	Education	Illness Duration (Years)	Diagnosis (Criteria)
**Adhia et al.** **2023 [105]**	15	39.2 ± 16.0	F: 9; M: 6	University degree: 8; trade/apprenticeship: 1; certificate/diploma: 2; Year 12/equivalent: 1; Year 10/equivalent: 3; no formal qualification: 0	7.4 ± 9.7	15	44.6 ± 13.1	F: 10; M: 5	University degree: 5; trade/apprenticeship: 1; certificate/diploma: 3; Year 12/equivalent: 2; Year 10/equivalent: 0; no formal qualification: 4	6.6 ± 5.4	Chronic low back pain (IASP guidelines, NPRS and Roland Morris disability questionnaire scales)
**Alcon et al.** **2025 [102]**	PNE + real tDCS: 10	46.8 ± 15.4	F: 10, M: 0	n.r.	5.97 ± 7.61	PNE + sham tDCS: 10	35.9 ± 17.4	F: 8, M: 2	n.r.	4.37 ± 3.6	Low back pain (European guidelines of non-specific chronic low back pain)
**Antal et al.** **2011 [93]**	13	33.2 ± 10.4	F: 12, M: 1	n.r.	15.3 ± 12.1	13	32.3 ± 12.3	F: 11, M: 2	n.r.	12.0 ± 8.9	Chronic migraine (IHS criteria—2004)
**Armbrust et al.** **2024 [103]**	OMT + tDCS: 24	38.25 ± 12.78	F: 15, M: 9	n.r.	n.r.	sham OMT + sham tDCS: 24; OMT + sham tDCS: 24	sham OMT + sham tDCS: 38.75 ± 15.34; OMT + sham tDCS: 35.50 ± 15.47	sham OMT + sham tDCS: F: 16, M: 8; OMT + sham tDCS: F: 18, M: 6	n.r.	n.r.	Chronic low back pain
**Arroyo-Fernandez et al.** **2022 [121]**	40	50.60 ± 7.01	F:38, M: 2	No studies:4; Primary: 12; Secondary: 7; High school: 3; Vocational Training: 7; University: 7	11.85 ± 7.30	40	49.53 ± 8.74	F: 39, M: 1	No studies:2; Primary: 16; Secondary: 6; High school: 4; Vocational Training: 10; University: 2	11.30 ± 6.59	Fibromyalgia (ACR 2010)
**Caumo et al.** **2022 [122]**	32	49.6 ± 9.00	F	11.33 ± 4.15	n.r.	16	48.0 ± 6.72	F	12.11 ± 4.04	n.r.	Fibromyalgia (ACR 2016)
**Caumo et al.** **2024 [123]**	real M1 hb-tDCS: 34; real DLPFC hb -tDCS: 34	real M1 hb-tDCS: 48.98 ± 10.08; real DLPFC hb -tDCS: 48.56 ± 8.93	F	real M1 hb-tDCS: 12.06 ± 4.92; real DLPFC hb -tDCS: 12.18 ± 4.68	n.r.	sham M1 hb-tDCS: 17; sham DLPFC hb -tDCS: 17	sham M1 hb -tDCS: 49.70 ± 8.97; sham DLPFC hb -tDCS: 40.61 ± 9.70	F	sham M1 hb-tDCS: 13.25 ± 4.17; sham DLPFC hb -tDCS: 13.11 ± 4.57	n.r.	Fibromyalgia (ACR 2016)
**de Melo et al.** **2020 [124]**	real LM1 5d tDCS: 11; real LM1 10d tDCS: 9	44.81 ± 8.8	F	n.r.	6.6 ± 5.38	sham LM1 5d tDCS: 11	n.a. ^a^	F	n.r. ^a^	n.a. ^a^	Fibromyalgia (ACR)
**de Paula et al.** **2023 [125]**	real LDN + real tDCS: 21; sham LDN + tDCS: 22	real LDN + real tDCS: 49.74 ± 1.97; sham LDN + real tDCS: 50.57 ± 2.23	F	real LDN + real tDCS: 10.00 ± 0.53; sham LDN + real tDCS: 13.00 ± 0.92	n.r.	real LDN + sham tDCS: 22; sham LDN + sham tDCS: 21	real LDN + sham t DCS: 48.09 ± 1.56; sham LDN + sham tDCS: 48.95 ± 2.08	F	real LDN + sham t DCS:11.55 ± 0.99; sham LDN + sham tDCS: 11.95 ± 0.87	n.r.	Fibromyalgia (ACR 2016)
**Dere et al.** **2025 [107]**	CSE + real tDCS: 10	31 ± 13.74	F: 9, M: 1	n.r.	n.r.	CSE + sham tDCS: 9; CSE: 10	CSE + sham tDCS: 33.22 ± 16.38; CSE: 35 ± 15.69	CSE + sham tDCS: F: 9, M: 0; CSE: F: 10, M: 0	n.r.	n.r.	Chronic neck pain (evaluated with VAS score)
**Dutra et al.** **2020 [89]**	13	26.1 ± 3.8	F	n.r.	n.r.	11	21.0 ± 2.1	F	n.r.	n.r.	Dysmenorrhea (Primary Dysmenorrhea Consensus Guideline)
**Ehsani et al.** **2023 [101]**	CBT + real tDCS: 15	33.00 ± 1.60 (SEM)	F: 8, M: 7	n.r.	n.r.	CBT + sham tDCS: 15; CBT: 15	CBT + sham tDCS: 33.00 ± 1.82 (SEM); CBT: 33.00 ± 1.77 (SEM)	CBT + sham tDCS: F: 8, M: 7; CBT: F: 7, M: 8	n.r.	n.r.	Chronic low back pain (temporal and VAS evaluation)
**Fagerlund et al.** **2015 [72]**	24	49.04 ± 8.63	F	n.r.	17.73 ± 7.54	24	48.17 ± 10.56	F: 21, M: 3	n.r.	18:50 ± 11.48	Fibromyalgia (ACR 1990)
**Hasırcı Bayır et al.** **2024 [94]**	Menstrual Migraine + real tDCS: 18; non-Menstrual Migraine + real tDCS: 11	n.r.	F	n.r.	n.r.	Menstrual Migraine + sham tDCS: 18; non-Menstrual Migraine + sham tDCS: 11	n.a. ^a^	F	n.a. ^a^	n.a. ^a^	Menstrual Migraine (ICHD-3 Criteria)
**Hazime et al.** **2017 [99]**	PES real + tDCS real: 23; PES sham + tDCS real: 23	PES real + tDCS real: 51.9 ± 9.9; PES sham + tDCS real: 51.3 ± 9.9	PES real + tDCS real: F: 18, M: 5; PES sham + tDCS real: F: 19, M: 4	PES real + tDCS real: Elementary school: 16, High school: 5, Higher Education: 2; PES sham + tDCS real: Elementary school: 12, High school: 9, Higher Education: 2	PES real + tDCS real: 3.11 ± 3.29; PES sham + tDCS real: 7.63 ± 9.03	PES real + tDCS sham: 23; PES sham + tDCS sham: 23	PES real + tDCS sham: 53.0 ± 9.9; PES sham + tDCS sham: 54.1 ± 9.8	PES real + tDCS sham: F: 15, M: 8; PES sham + tDCS sham: F: 17, M: 6	PES real + tDCS sham: Elementary school: 11, High school: 10, Higher Education: 2; PES sham + tDCS sham: Elementary school: 18, High school: 4, Higher Education: 1	PES real + tDCS sham: 4.98 ± 4.98; PES sham + tDCS sham: 5.77 ± 7.73	Non-specific low back pain (medical diagnosis)
**Hervik et al.** **2024 [95]**	20	48.6 ± 4.4	F: 14, M: 6	n.r.	n.r.	20	46.3 ± 6.1	F: 17, M: 3	n.r.	n.r.	Primary chronic headaches (IHS criteria—2020)
**Hodaj et al.** **2022 [92]**	18	54.5 ± 10.6	F: 10, M: 8	n.r.	27.8 ± 13.5	18	46.1 ± 14.1	F: 16, M:2	n.r.	24.1 ± 15.7	Chronic migraine (IHS criteria—2018)
**Jiang et al.** **2020 [100]**	26	39.9 ± 14.2	F: 12, M: 14	n.r.	2.1 ± 4.1	25	44.1 ± 13	F: 12, M: 13	n.r.	2.5 ± 3.9	Low back pain (medical decision)
**Jornada et al.** **2024 [126]**	real M1 hb-tDCS: 34; real DLPFC hb-tDCS: 34	real M1 hb-tDCS: 48.98 ± 10.08; real DLPFC hb-tDCS: 48.56 ± 8.93	F	real M1 hb-tDCS: 12.06 ± 4.92; real DLPFC hb-tDCS: 12.18 ± 4.68	n.r.	sham M1 hb-tDCS: 17; sham DLPFC hb-tDCS: 17	sham M1 hb-tDCS: 49.70 ± 8.97; sham DLPFC hb-tDCS: 40.61 ± 9.70	F	sham M1 hb-tDCS: 13.25 ± 4.17; sham DLPFC hb-tDCS: 13.11 ± 4.57	n.r.	Fibromyalgia (ACR 2016)
**Khedr et al.** **2017 [86]**	18	31.3 ± 10.99	F: 17, M: 1	n.r.	0.51 ± 0.22	18	33.89 ± 11.18	F: 17, M: 1	n.r.	0.50 ± 0.21	Fibromyalgia (ACR 2010)
**Lin et al.** **2022 [127]**	19	48.3 ± 13.6	F: 13, M: 6	n.r.	n.r.	19	48.9 ± 12.3	F: 17, M: 2	n.r.	n.r.	Fibromyalgia (ACR 2016)
**Lopes Alves et al.** **2024 [128]**	hb real DLPFC tDCS: 16; hb real M1 tDCS: 13	hb real DLPFC tDCS: 48.75 ± 9.04; hb real M1 tDCS: 49.38 ± 11.54	F	hb real DLPFC tDCS: 12.00 ± 3.83; hb real M1 tDCS: 11.62 ± 3.79	n.r.	hb sham DLPFC tDCS: 8; hb sham M1 tDCS: 6	hb sham DLPFC tDCS: 45.88 ± 10.41; hb sham M1 tDCS: 44.17 ± 9.06	F	hb sham DLPFC tDCS: 12.50; hb sham M1 tDCS: 13.83 ± 3.31	n.r.	Fibromyalgia (according to the ACR criteria of 2016)
**Loreti et al.** **2023 [129]**	17	42.82 ± 9.02	F	Illiterate (5.88%), 1 to 4 (11.76%), 5 to 8 (17.66%), 9 to 11 (52.94%), ≥12 (11.76%)	5.47 ± 2.63	18	41.17 ± 11.31	F	Illiterate (0%), 1 to 4 (16.66%); 5 to 8 (27.77%), 9 to 11 (33.35%), ≥12 (22.22%)	5.83 ± 3.33	Fibromyalgia (ACR 2011)
**Luedtke et al.** **2015 [73]**	67	45 ± 9	F:33, M:34	n.r.	8.2 ± 8.8	68	44 ± 10	F:30, M:38	n.r.	7.8 ± 10.4	Low back pain (European guidelines of non-specific chronic low back pain)
**Matias et al.** **2022 [130]**	17	48.94 ± 13.83	F	Elementary: 23.53%, Secondary: 64.7, University: 11.76	n.r.	14	49.43 ± 15.14	F	Elementary: 21.42%, Secondary: 71.4, University: 7.14	n.r.	Fibromyalgia (ACR 2010)
**Mechsner et al.** **2023 [131]**	18	32.1 ± 9.03	F	n.r.	3.57 ± 3.7	18	29.1 ± 7.30	F	n.r.	3.54 ± 2.5	Chronic pelvic pain and endometriosis (medical records or medical evaluation)
**Oliveira et al.** **2015 [96]**	16	23.8 ± 7.3	F: 15, M: 1	High school: 4, incomplete college degree: 10, college degree: 2	2.5 ± 1.4	16	25.5 ± 6.3	F: 14, M: 2	High school: 5, incomplete college degree: 8, college degree: 3	2.8 ± 1.9	Temporomandibular Disorder (TMD) (DCR)
**Ramasawmy et al.** **2024 [132]**	hb real tDCS + MM: 18	51.8 ± 2.78	F: 20, M: 2	n.r.	n.r.	hb sham tDCS + MM: 19	51.6 ± 2.66	F: 21, M: 1	n.r.	n.r.	Fibromyalgia (ACR 2010)
**Riberto et al.** **2011 [88]**	11	58.3 ± 12.1	F	6.3 ± 5.2	0.83 ± 0.98	12	52.4 ± 11.5	F	8.9 ± 4.4	0.5 ± 0.9	Fibromyalgia (ACR 1990)
**Samartin-Veiga et al.** **2022 [71]**	M1: 34; DLPFC: 33; OIC: 33	M1: 49.38 ± 8.83; DLPFC: 50.55 ± 8.89; OIC: 50.21 ± 8.20	F	n.r.	n.r.	30	50.67 ± 8.88	F	n.r.	n.r.	Fibromyalgia (ACR 2010)
**Segal et al.** **2021 [106]**	MT + real tDCS: 10	58.1 ± 10.9	F: 7, M: 23	n.r.	n.r.	MT + sham tDCS: 10; MT: 10	n.a. ^a^	n.a. ^a^	n.a. ^a^	n.a. ^a^	Phantom limb (mean daily phantom pain intensity ≥ 4)
**Serrano et al.** **2022 [133]**	24	49.18 ± 8.63	F	10.23 ± 3.75	n.r.	12	46.09 ± 11.34	F	12.64 ± 5.07	n.r.	Fibromyalgia (ACR 2016)
**Sornkaew et al.** **2024 [104] **	MCE + real tDCS: 12	29.2 ± 7.0	F: 7, M: 5	n.r.	3.63 ± 3.92	MCE + sham tDCS: 10	29.8 ± 8.0	F: 5, M: 5	n.r.	2.93 ± 4.48	Chronic low back pain
**To et al.** **2017 [134]**	OC: 15; FC: 11	OC: 47.13 ± 10.01; FC: 47.81 ± 10.17	OC: F:12, M: 3; FC: F: 10, M: 1	n.r.	n.r.	16	n.r.	F: 14, M: 2	n.r.	n.r.	Fibromyalgia (ACR 1990)
**Volz et al.** **2016 [108]**	10	40.6 ± 12.5	F: 7, M: 3	n.r.	10 ± 8.9	10	34.4 ± 13.2	F: 6, M: 4	n.r.	7 ± 4.7	Inflammatory bowel syndrome (medical report, discharge letter, and outpatient center records)

Notes: ^a^ = Mean divided by condition not available; 5d = five day; 10d = ten day; ACR = American College of Rheumatology; CBT = Cognitive Behavioral Therapy; CSE: Cervical Stabilization Exercises; DCR = Diagnostic Criteria for Research; DLPFC = dorsolateral prefrontal cortex; F = female; FC = frontal cortex; hb = home-based; IASP = International Association for the Study of Pain; ICHD-3 stands for the International Classification of Headache Disorders, 3rd edition; IHS = International Headache Society; LDN = Low-Dose Naltrexone; LM1 = left primary motor cortex; M = male; M1 = primary motor cortex; MCE = Motor Control Exercise; MM = Mindfulness Meditation; MT = Mirror Therapy; N = number; n.a. = not applicable; NPRS = Numeric Pain Rating Scale; n.r. = not reported; OC = occipital cortex; OMT = Osteopathic Manipulative Treatment; PES = Peripheral Electrical Stimulation; PNE = Pain Neuroscience Education; SEM = Standard Error of the Mean; tDCS = transcranial direct current stimulation; tES = transcranial electric stimulation; VAS = Visual Analogue Scale.

**Table 3:** Qualitative data on TMS protocols used in the included studies.

Study	Sessions Number	Frequency and Intensity ^a^	Pulses Number	Duration (Minutes)	Protocol Type	Coil Type	Target Region	Therapeutic Strategy ^1^
**AbdElkader et al. 2021 [91]**	12	5 Hz, 90%	1000	5	Excitatory	Figure-of-8 coil	LDLPFC	n.r.
**Altas et al. 2019 [87]**	15	10 Hz, 90%	1200	30	Excitatory	Figure-of-8 coil	(i) LM1, (ii) LDLPFC	Add-on
**Avery et al. 2015 [90]**	15	10 Hz, 120%	3000	37.5	Excitatory	Not specified	LDLPFC	Add-on
**Badr et al. 2024 [112]**	20	1 Hz, 120%	1200	30	Inhibitory	Figure-of-8 coil	RDLPFC	Add-on
**Bilir et al. 2021 [74]**	14	10 Hz, 90%	1500	15	Excitatory	Figure-of-8 coil	LDLPFC	Add-on
**Boyer et al. 2014 [113]**	14	10 Hz, 90%	2000	20	Excitatory	Figure-of-8 coil	LM1	Add-on
**Guinot et al. 2021 [114]**	16	10 Hz, 80%	2000	20	Excitatory	Not specified	Dominant M1	Augmentative
**Herrero Babiloni et al. 2024 [97]**	1	20 Hz, 80%	1500	20	Excitatory	Double 70 mm AirFilm R Coil	M1 of most painful represented painful site	Add-on
**Joshi et al. 2024 [111]**	10	10 burst (3 pulses per 50 Hz), 20 trains, 80%	600	40 s	Excitatory	Figure-of-8 coil	(i) LM1, (ii) DLPFC	Add-on
**Kankane et al., 2024 [115]**	10	(i) 10 Hz, 80% (ii) 1 Hz, 80%	(i) 10 per train; (ii) 150 per train	(i) 20 (ii) 27	(i) Excitatory (ii) Inhibitory	Butterfly coil	(i) LDLPFC, (ii) RDLPFC	Add-on
**Mhalla et al. 2011 [116]**	14	10 Hz, 80%	1500	15	Excitatory	Figure-of-8 coil	LM1	Add-on
**Passard et al. 2007 [117]**	10	10 Hz, 80%	2000	25	Excitatory	Figure-of-8 coil	LM1	Add-on
**Picarelli et al. 2010 [109]**	10	10 Hz, 100%	2500	30	Excitatory	Figure-of-8 coil	M1	Add-on
**Tekin et al. 2014 [118]**	10	10 Hz, 100%	1500	10	Excitatory	Figure-of-8 coil	LM1	Monotherapy
**Tilbor et al. 2024 [119]**	20	20 Hz, 100%	2000	10 ca	Excitatory	H7 coil	ACC, mPFC	Augmentative
**Todorov et al. 2020 [70]**	5	15 Hz, 70%	1200	8	Excitatory	Figure-of-8 coil	(i) M1, (ii) LDLPFC	Add-on
**Umezaki et al. 2016 [98]**	10	10 Hz, 110%	3000	15	Excitatory	Figure-of-8 coil	LDLPFC	Add-on
**Vanhanen et al. 2023 [110]**	10	10 Hz, 90%	1500	8 ca	Excitatory	Figure-of-8 coil	S2	Add-on
**Yağci et al. 2014 [120]**	10	1 Hz, 90%	1200	15	Inhibitory	Parabolic coil	LM1	Add-on

Notes: ^1^ monotherapy = when patients did not receive additional medications or treatments to NIBS; Add-on = when NIBS is added to existing interventions (i.e., pharmacotherapy); Augmentative approach = when other pharmacological, behavioral, or psychological interventions are experimentally combined to the stimulation protocol [35]. ^a^ Intensity is reported as a percentage of the resting motor threshold. Notes: ACC = anterior cingulate cortex; DLPFC = dorsolateral prefrontal cortex; Hz = Hertz; LDLPFC = left DLPFC; LM1 = left primary motor cortex; M1 = primary motor cortex; mm = millimeters; mPFC = medial prefrontal cortex; n.r. = not reported; RDLPFC = right LDLPFC = left dorsolateral prefrontal cortex; S2 = secondary somatosensory cortex.

**Table 4:** Qualitative data on tES protocols used in the included studies.

Study	Sessions Number	tES	Stimulation Intensity (mA)	Stimulation Intensity Density (j)	Duration (Minutes)	Target Region	Electrodes Montage ^a^ (Anode—Cathode)	Protocol Type	Electrode Size	Therapeutic Strategy ^1^
**Adhia et al. 2023 [105]**	1	HD-tIPNS	2 mA	0.5	30	pgACC, dACC, and S1	CP1, CP2, F3, F4, F7, OZ, T8, Cz	n.r.	4 cm^2^	Monotherapy
**Alcon et al. 2025 [102]**	5	tDCS	2	0.08	20	LDLPFC	F3—right forehead	Excitatory	25 cm^2^	Augmentative
**Antal et al. 2011 [93]**	9	tDCS	1	0.03	15	OC	CZ-OZ	Inhibitory	35 cm^2^	Add-on
**Armbrust et al. 2024 [103]**	10	tDCS	2	0.08	20	M1	C3 or C4 dominant hemispere-Fp2	Excitatory	25 cm^2^	Augmentative
**Arroyo-Fernandez et al. 2022 [121]**	5	tDCS	2	0.08	20	LM1	C3-Fp2	Excitatory	25 cm^2^	Augmentative
**Caumo et al. 2022 [122]**	20	tDCS	2	0.06	20	DLPFC	F3-F4	Bihemispheric	35 cm^2^	Add-on
**Caumo et al. 2024 [123]**	20	hb-tDCS	2	0.06	20	(i) LM1; (ii) DLPFC	(i) C3–Fp2; (ii)F3–F4	(i) Excitatory; (ii) Bihemispheric	35 cm^2^	Add-on
**de Melo et al. 2020 [124]**	5	tDCS	2	0.06	20	LM1	C3–Fp2	Excitatory	35 cm^2^	Add-on
**de Paula et al. 2023 [125]**	5	tDCS	2	0.06	20	M1	anode: M1 contralateral to the dominant cortex—Fp1 or Fp2	Excitatory	35 cm^2^	Augmentative
**Dere et al. 2025 [107]**	16	tDCS	2	0.06	20	M1	C3 or C4—Fp2 or Fp1 contralateral site	Excitatory	35 cm^2^	Augmentative
**Dutra et al. 2020 [89]**	5	tDCS	2	0.06	20	LDLPFC	F3-Fp2	Excitatory	35 cm^2^	Monotherapy
**Ehsani et al. 2023 [101]**	8	tDCS	2	0.06	20	LDLPFC	contralateral supraorbital area-F3	Inhibitory	35 cm^2^	Augmentative
**Fagerlund et al. 2015 [72]**	5	tDCS	2	0.06	20	LM1	C3-Fp2	Excitatory	35 cm^2^	Add-on
**Hasırcı Bayır et al. 2024 [94]**	3	tDCS	2	0.06	20	LDLPFC	F3—Fp2	Excitatory	35 cm^2^	Add-on
**Hazime et al. 2017 [99]**	12	tDCS	2	0.06	20	M1	cpM1- Fp2/Fp1 ^b^	Excitatory	35 cm^2^	Augmentative
**Hervik et al. 2024 [95]**	8	tDCS	2	0.06	30	LM1	LM1—controlateral supraorbital area	Excitatory	35 cm^2^	Add-on
**Hodaj et al. 2022 [92]**	5	tDCS	2	0.06	20	LM1	C3-Fp2	Excitatory	35 cm^2^	Add-on
**Jiang et al. 2020 [100]**	1	tDCS	2	0.25/0.5	20	M1	C3 or C4 contralateral painful area Fp1/Fp2 ^b^	Excitatory	Round electrodes (anode/cathode diameter: 1 cm/4 cm)	Monotherapy
**Jornada et al. 2024 [126]**	20	hb-tDCS	2	0.06	20	(i) LM1; (ii) LDLPFC	(i) C3—Fp2; (ii) F3—F4	(i) Excitatory; (ii) Bihemispheric	35 cm^2^	Add-on
**Khedr et al. 2017 [86]**	10	tDCS	2	0.08	20	LM1	C3-right arm	Excitatory	24 cm^2^	n.r.
**Lin et al. 2022 [127]**	20	tACS	Monophasic square wave with a pulse width of 0.5 ms and intensity of 1 mA at 50 Hz, repeated at duty cycle with on time of 2 s and off time of 8 s	cannot be calculated	20	LM1	C3-CZ, F3, T7, P3	Excitatory	4 × 1 ring electrode configuration	Add-on
**Lopes Alves et al. 2024 [128]**	20	hb-tDCS	2	0.06	20	(i) LM1; (ii) DLPFC	(i) LM1—Fp2; (ii) LDLPFC-RDLPFC	(i) Excitatory; (ii) Bihemispheric	35 cm^2^	Add-on
**Loreti et al. 2023 [129]**	10	tDCS	2	0.06	26	LM1	C3—right supraorbital region	Excitatory	35 cm^2^	n.r.
**Luedtke et al. 2015 [73]**	5	tDCS	2	0.06	20	LM1	C3-Fp2	Excitatory	35 cm^2^	Add-on
**Matias et al. 2022 [130]**	5	tDCS	2	0.06	20	LM1	C3-Fp2	Excitatory	35 cm^2^	Augmentative
**Mechsner et al. 2023 [131]**	10	tDCS	2	0.06	20	M1 dominant hemisphere	C3 or C4—Fp2 or Fp1 contralateral site	Excitatory	35 cm^2^	Add-on
**Oliveira et al. 2015 [96]**	5	tDCS	2	0.06	20	M1	cpM1-Fp1/Fp2 ^b^	Excitatory	35 cm^2^	Augmentative
**Ramasawmy et al. 2024 [132]**	10	HB tDCS	2	0.08	20	LM1	C3—Fp2	Excitatory	25 cm^2^	Augmentative
**Riberto et al. 2011 [88]**	10 once a week	tDCS	2	0.06	20	LM1	C3-Fp2	Excitatory	35 cm^2^	Augmentative
**Samartin-Veiga et al. 2022 [71]**	15	tDCS	M1 and DLPFC: 2 OIC-MULTIELECTRODE F3 = 0.565; FC1 = 0.508; F8 = 0.158; FC5 = 0.579; C5 = 1.144; P3 = 0.492	M1 & DLPFC: 0.08 OIC: 0.6 distributed over the multielectrode montage	20	(i) LM1; (ii) LDLPFC; (iii) OIC	(i) M1 C3-Fp2; (ii) DLPFC F3-Fp2; (iii) OIC-MULTIELECTRODE F3, FC1, F8- FC5, C5, P3	(i)–(ii) M1/DLPFC Excitatory; (iii) OIC Multifocal	(i)–(ii) M1 & DLPFC 25 cm^2^; (iii) OIC 3.14 cm^2^	Add-on
**Segal et al. 2021 [106]**	10	tDCS	1.5	0.4	22	M1	M1-contralateral forehead	Excitatory	35 cm^2^	Augmentative
**Serrano et al. 2022 [133]**	20	tDCS	2	0.06	20	LDLPFC	F3-F4	Bihemispheric	35 cm^2^	Add-on
**Sornkaew et al. 2024 [104]**	12	tDCS	2	0.06	20	M1 contralateral to painful side	M1 contralateral to painful side -Fp1/Fp2	Excitatory	35 cm^2^	Augmentative
**To et al. 2017 [134]**	8	tDCS	1.5	0.4	20	(i) OCC; (ii) DLPFC	(i) OCCIPITAL left C2 nerve dermatome—right C2 nerve dermatome; (ii) Bihemispheric F4-F3	(i) OCCIPITAL Excitatory; (ii) DLPFC Bihemispheric	35 cm^2^	Add-on
**Volz et al. 2016 [108]**	5	tDCS	2	0.06	20	M1	cpM1- Fp1/Fp2 ^b^	Excitatory	35 cm^2^	Add-on

Notes: ^1^ monotherapy = when patients did not receive additional medications or treatments; Add-on = when added to existing interventions (i.e., pharmacotherapy); Augmentative approach = when other pharmacological, behavioral, or psychological interventions were combined to the stimulation protocol [35]. ^a^ Electrode montage according to the EEG 10-20 system [144]. ^b^ Fp1 or Fp2 left or right supraorbital region. Notes: caM1 = contralateral to the amputated limb M1; cm^2^ = square centimeters; cpM1 = M1 contralateral to painful side of the body; dACC = dorsal anterior cingulate cortex; DLPFC = dorsolateral prefrontal cortex; hb = home-based; HD-tIPNS = High-Definition Transcranial Infraslow Pink-Noise Stimulation; Hz = Hertz; LDLPFC = left DLPFC; LM1 = left M1; M1 = primary motor cortex; mA = milliampere; OC = occipital cortex; OIC = Operculo–Insular Cortex; pgACC = pregenual anterior cingulate cortex; RDLPFC = right DLPFC; S1 = primary somatosensory cortex; tACS = transcranial Alternate Current Stimulation; tDCS = transcranial direct current stimulation.

## Data Availability

No new data were created or analyzed in this study.

## Supplementary Materials

The following supporting information can be downloaded at https://www.mdpi.com/article/10.3390/brainsci16070663/s1. References used in the Supplementary Materials are cited here for completeness [197,198,199,200,201,202,203,204,205,206,207,208,209,210]. Table S1: The table shows details on the search strategy in the screened databases; Table S2: The table summarizes the location of patient recruitment; Table S3: The table summarizes protocol features from TMS studies; Table S4: The table summarizes additional protocol features of tES studies included in the meta-analysis; Table S5: Models’ selection; Table S6: Subgroup analysis of the pre–post pain intensity change score; Table S7: Covariate analysis of the pre–post pain intensity change score; Table S8: Subgroup analysis in pre–post emotional distress change score; Table S9: Covariate analysis of the pre–post emotional distress change score; Table S10: Subgroup analysis in pre–post functional disability change score; Table S11: Subgroup analysis in pre–post functional disability change score; Table S12: Subgroup analysis of the pre-follow-up pain intensity change score; Table S13: Meta-regression analysis of the pre-follow-up pain intensity change score; Table S14: Subgroup analysis of the pre-follow-up emotional distress change score; Table S15: Covariate analysis of the pre-follow-up emotional distress change score; Table S16: Subgroup analysis in pre-follow-up functional disability change score; Table S17: Subgroup analysis in pre-follow-up functional disability change score; Figure S1: Forest plot of the effect size of NIBS on pain intensity change score at follow-up; Figure S2: Baujat plot of study contributions to heterogeneity for pain intensity at follow-up; Figure S3: Forest plot of the effect size of NIBS on emotional distress change score at follow-up; Figure S4: Baujat plot of study contributions to heterogeneity for emotional distress at follow-up; Figure S5: Forest plot of the effect size of NIBS functional disability change score at follow-up; Figure S6: Baujat plot of study contributions to heterogeneity for functional disability at follow-up; Figure S7: Forest plot of the effect size of noninvasive brain stimulation on quality-of-life change score; Figure S8: Baujat plot of study contributions to heterogeneity for quality of life as the outcome measure; PRISMA 2020 checklist.

## Abbreviations

The following abbreviations are used in this manuscript:

CPP	Chronic primary pain
NIBS	Non-invasive brain stimulation
TMS	Transcranial magnetic stimulation
rTMS	Repetitive transcranial magnetic stimulation
tDCS	Transcranial direct current stimulation
tES	Transcranial electric stimulation
