# Supplementary material for: Non-Invasive Brain Stimulation for Core Symptoms of Chronic Primary Pain: A Meta-Analysis of RCTs
Source: Brain Sci. 2026 Jun 24;16(7):663. doi: 10.3390/brainsci16070663 (PMC13406848; doi:10.3390/brainsci16070663)
Supplement: Supplementary file 1 [file brainsci-16-00663-s001.zip › brainsci-4320322-supplementary.pdf]

Non-invasive Brain Stimulation for Core Symptoms of Chronic Primary Pain: A Meta-Analysis of RCTs

Telesca A.*, Vergallito A.*, Vedani A., Locatelli G., Visiello B., Romero Lauro L.J.

Supplementary materials

Section A – Methods supplementary information

Study quality assessment

Two authors (A.T., A.Ved.) performed quality assessment independently using the Cochrane Collaboration's Risk-of-Bias Tool for RCT [1] which focuses on five domains: *1) Bias arising from the randomization process,* assessing whether the allocation sequence was properly generated and concealed to prevent selection bias; *2) bias due to deviations from intended interventions*, which evaluates whether participants and personnel were blinded to the interventions and whether any deviations from the intended interventions occurred*; 3) bias due to missing outcome data*, assessing the completeness of outcome data and whether any missing data could affect the results; 4) *bias in the measurement of the outcomes,* evaluating how outcomes were measured and whether knowledge of the intervention by outcome assessors could have influenced the results; 5) *bias in the selection of the reported results*, assessing whether outcomes were selectively reported (i.e., including only positive findings or omitting non-significant results). Conflicts were resolved by consensus between the two researchers, and a third researcher was consulted when necessary (A.Ver.).

Search strategy

Table S1. The table shows details on the search strategy in the screened databases.	
**Database**	**Search strings**
Pubmed (Feb 2025)	(("atypical facial pain"[Title/Abstract] OR "burning mouth syndrome"[Title/Abstract] OR "burning mouth syndrome"[MeSH Terms] OR "migraine disorders"[Title/Abstract] OR "migraine disorders"[MeSH Terms] OR "abdominal pain"[Title/Abstract] OR "abdominal pain"[MeSH Terms] OR "neck pain"[Title/Abstract] OR "neck pain"[MeSH Terms] OR "chest pain"[Title/Abstract] OR "chest pain"[MeSH Terms] OR "phantom limb"[Title/Abstract] OR "phantom limb"[MeSH Terms] OR "low back pain"[Title/Abstract] OR "low back pain"[MeSH Terms] OR "facial pain"[Title/Abstract] OR "facial pain"[MeSH Terms] OR "pelvic pain"[Title/Abstract] OR "pelvic pain"[MeSH Terms] OR "visceral pain"[Title/Abstract] OR "visceral pain"[MeSH Terms] OR "temporomandibular joint dysfunction syndrome"[Title/Abstract] OR "temporomandibular joint dysfunction syndrome"[MeSH Terms] OR "tension-type headache"[Title/Abstract] OR "tension-type headache"[MeSH Terms] OR "complex regional pain syndromes"[Title/Abstract] OR "complex regional pain syndromes"[MeSH Terms] OR "fibromyalgia"[Title/Abstract] OR "fibromyalgia"[MeSH Terms] OR "irritable bowel syndrome"[MeSH Terms] OR "irritable bowel syndrome"[Title/Abstract] OR "persistent idiopathic facial pain"[Title/Abstract] OR "small-fiber neuropathy"[Title/Abstract] OR "small-fiber neuropathy"[MeSH Terms] OR "trigeminal autonomic cephalalgias"[Title/Abstract] OR "trigeminal autonomic cephalalgias"[MeSH Terms] OR "trigeminal neuralgia"[Title/Abstract] OR "trigeminal neuralgia"[MeSH Terms] OR "interstitial cystitis"[Title/Abstract] OR "Hypertrophic Gastritis"[Title/Abstract] OR "Hypertrophic Gastritis"[MeSH Terms] OR "Prostatitis"[Title/Abstract] OR "Prostatitis"[MeSH Terms] OR "idiopathic pain"[Title/Abstract] OR "primary pain"[Title/Abstract])) AND ("transcranial magnetic stimulation"[MeSH Terms] OR "transcranial magnetic stimulation"[Title/Abstract] OR "tms"[Title/Abstract] OR "transcranial direct current stimulation"[Title/Abstract] OR "transcranial direct current stimulation"[MeSH Terms] OR "tdcs"[Title/Abstract])
Scopus (Feb 2025)	(TITLE-ABS-KEY ("atypical facial pain") OR TITLE-ABS-KEY ("chronic burning mouth") OR TITLE-ABS-KEY ("chronic migraine") OR TITLE-ABS-KEY ("chronic primary abdominal pain syndrome") OR TITLE-ABS-KEY ("chronic primary bladder pain syndrome") OR TITLE-ABS-KEY ("chronic primary cervical pain") OR TITLE-ABS-KEY ("chronic primary chest pain syndrome") OR TITLE-ABS-KEY ("chronic primary epigastric pain syndrome") OR TITLE-ABS-KEY ("Chronic primary limb pain") OR TITLE-ABS-KEY ("chronic primary low back pain") OR TITLE-ABS-KEY ("chronic primary orofacial pain") OR TITLE-ABS-KEY ("chronic primary pelvic pain syndrome") OR TITLE-ABS-KEY ("chronic tension-type headache") OR TITLE-ABS-KEY ("chronic temporomandibular disorder pains") OR TITLE-ABS-KEY ("complex regional pain syndromes") OR TITLE-ABS-KEY ("fibromyalgia") OR TITLE-ABS-KEY ("irritable bowel syndrome") OR TITLE-ABS-KEY ("persistent idiopathic facial pain") OR TITLE-ABS-KEY ("small-fiber neuropathy") OR TITLE-ABS-KEY ("trigeminal autonomic cephalalgias") OR TITLE-ABS-KEY ("trigeminal neuralgia") OR TITLE-ABS-KEY ("chronic primary thoracic pain") OR TITLE-ABS-KEY ("chronic primary visceral pain") OR TITLE-ABS-KEY ("interstitial cystitis") OR TITLE-ABS-KEY ("idiopathic pain") OR TITLE-ABS-KEY ("primary pain")) AND (TITLE-ABS-KEY ("transcranial magnetic stimulation") OR TITLE-ABS-KEY ("tms") OR TITLE-ABS-KEY ("transcranial direct current stimulation") OR TITLE-ABS-KEY ("tdcs") OR TITLE-ABS-KEY ("repetitive transcranial magnetic stimulation") OR TITLE-ABS-KEY ("rtms"))
Embase (Feb 2025)	('atypical facial pain'/exp OR 'atypical facial pain' OR 'burning mouth syndrome':ti,ab,kw OR 'migraine'/exp OR 'migraine' OR 'abdominal pain'/exp OR 'abdominal pain' OR 'neck pain':ti,ab,kw OR 'chest pain':ti,ab,kw OR 'phantom limb'/exp OR 'phantom limb' OR 'low back pain':ti,ab,kw OR 'face pain'/exp OR 'face pain' OR 'pelvic pain'/exp OR 'pelvic pain' OR 'visceral pain'/exp OR 'visceral pain' OR 'temporomandibular joint disorder'/exp OR 'temporomandibular joint disorder' OR 'tension headache'/exp OR 'tension headache' OR 'complex regional pain syndrome'/exp OR 'complex regional pain syndrome' OR 'fibromyalgia'/exp OR 'fibromyalgia' OR 'irritable bowel syndrome':ti,ab,kw OR 'persistent idiopathic facial pain'/exp OR 'persistent idiopathic facial pain' OR 'small-fiber neuropathy'/exp OR 'small-fiber neuropathy' OR 'trigeminal autonomic cephalalgia'/exp OR 'trigeminal autonomic cephalalgia' OR 'trigeminal neuralgia':ti,ab,kw OR 'interstitial cystitis'/exp OR 'interstitial cystitis' OR 'menetrier disease'/exp OR 'menetrier disease' OR 'prostatitis'/exp OR 'prostatitis' OR 'idiopathic pain'/exp OR 'idiopathic pain' OR 'primary pain':ti,ab,kw) AND ('transcranial magnetic stimulation'/exp OR 'transcranial

	magnetic stimulation' OR 'tms'/exp OR 'tms' OR 'transcranial direct current stimulation'/exp OR 'transcranial direct current stimulation' OR 'tdcs')
Web of Science (Feb 2025)	TS=('atypical facial pain' OR 'chronic burning mouth' OR 'chronic migraine' OR 'chronic primary abdominal pain syndrome' OR 'chronic primary bladder pain syndrome' OR 'chronic primary cervical pain' OR 'chronic primary chest pain syndrome' OR 'chronic primary epigastric pain syndrome' OR 'chronic primary limb pain' OR 'chronic primary low back pain' OR 'chronic primary orofacial pain' OR 'chronic primary pelvic pain syndrome' OR 'chronic tension-type headache' OR 'chronic temporomandibular disorder pains' OR 'complex regional pain syndromes' OR 'fibromyalgia' OR 'irritable bowel syndrome' OR 'persistent idiopathic facial pain' OR 'small-fiber neuropathy' OR 'trigeminal autonomic cephalalgias' OR 'trigeminal neuralgia' OR 'chronic primary thoracic pain' OR 'chronic primary visceral pain' OR 'interstitial cystitis' OR 'idiopathic pain' OR 'primary pain') AND TS=('transcranial magnetic stimulation' OR 'tms' OR 'repetitive transcranial magnetic stimulation' OR 'rtms' OR 'transcranial direct current stimulation' OR 'tdcs')

Outcomes measures' details

Studies varied in the outcome measures collected, typically including self-administered questionnaires to assess pain severity, emotional distress, the functional impact of the disease, and quality of life.

Pain severity was typically measured using the Visual Analog Scale (VAS) and the Numerical Rating Scale (NRS). Papers reporting only a measure of pain frequency to indicate pain severity [2,3] were not included in the analysis. Indeed, frequency is typically considered a measure of pain for migraines but not for other CPP conditions. Results considering the effects of NIBS on the frequency of migraine attacks can be found in previous meta-analyses [2,4,5].

E*motional distress* was typically measured through clinicians-administered or self-report standardized questionnaires investigating anxiety and depressive symptoms, such as the Hospital Anxiety and Depression Scale (HADS) [6], the Beck Anxiety Inventory (BAI) [7], the Beck Depression Inventory (BDI) [8], the Hamilton Depression Rating Scale (HDRS) [9], the Hamilton Anxiety Rating Scale (HARS) [10], the Montgomery-Asberg Depression Rating Scale (MADRS) [11], and the State-Trait Anxiety Inventory (STAI) [12].

F*unctional disability* was typically investigated through the Roland-Morris Disability Questionnaire (RMDQ) [13] or the Oswestry Disability Index (ODI) [13]. Functional disability refers to a person's ability to perform daily activities, such as walking and dressing, or participate in social or work-related roles [14]. Quality of life is a broader and more subjective measure that encompasses physical functionality and emotional, psychological, and social well-being [15,16], thereby reflecting the overall level of satisfaction individuals experience in their lives.

The quality of life was assessed using standardized questionnaires, such as the Short Form Health Survey 36 (SF-36) [17-23], 12-item Short-Form Health Survey questionnaire (SF-12) [3,24], RAND 36-item health survey (RAND-36) [25,26], the Inflammatory Bowel Disease Questionnaire (IBDQ) [27,28], EuroQoL 5-dimension 3-level (EQ-5D-3L) [29,30], and the World Health Organization Quality of Life Scale – Brief version (WHOQOL-BRIEF) [31-34]. It is crucial to highlight that, since the IASP definition of CPP is relatively recent, the included papers varied in their inclusion (or non-inclusion) of previous outcome measures. Therefore, considering the aims of our work, one of the authors (A.T.) categorized the scales collected in each paper into the three core symptoms according to the construct measured by each questionnaire.

Section B – Qualitative synthesis results of the included RCTs

Overall outcome measures

In this systematic review, the effects of NIBS in CPP were measured on the three core symptoms of the disease [14], namely pain intensity, emotional distress, and functional disability. Since some articles assessed participants' quality of life, we also explored NIBS's effects on this measure. Since the CPP diagnosis is recent, not all studies have addressed all three core symptoms. Pain intensity was the primary outcome in most included studies, whereas emotional distress and functional disability were assessed in fewer studies.

None of the included studies directly investigated patients with a CPP diagnosis; rather, they focused on specific chronic pain conditions that are currently classified as CPP. Consequently, one of the authors (A.T.) decided whether patients from each paper should be considered under the CPP diagnosis. Of the 19 studies applying TMS, 17 assessed pain intensity, 12 included measures of emotional distress, and 12 also assessed functional disability. Only four investigated the quality of life.

Among the 35 studies that applied tES, 27 assessed pain intensity, 17 assessed emotional distress, 23 assessed functional disability, and 9 assessed quality of life.

Section C: Supplementary details on patients and NIBS protocols

Details on patients' recruitment

Table S2. The table summarizes the location of patient recruitment.	
**TMS protocols**	
Authors	***Place of recruitment***
Ali AbdElkader et al. 2021	Outpatient Clinic
Altas et al. 2019	Physical Medicine And Rehabilitation
Avery et al. 2015	Advertising, Publicity On Television, And A Specialized Hospital Clinic
Badr et al. 2024	Tanta And Assiut University Hospitals
Bilir et al. 2021	Physical Medicine And Rehabilitation Outpatient Clinics
Boyer et al. 2014	La Timone University Hospital (Marseille, France)
Guinot et al. 2021	Pain And Rheumatology Department
Herrero Babiloni et al. 2024	Hôpital Du Sacré-Cœur De Montréal Within The CIUSSS Du Nord-De-l'Île-De-Montréal
Joshi et al. 2024	Department Of Psychiatry Of A Tertiary Care Hospital In North India Hospital
Kankane et al. 2024	Department Of Neurology And Psychiatry At Maharani Laxmi Bai (MLB) Medical College, Jhansi, Uttar Pradesh, India
Mhalla et al. 2011	N.R.
Passard et al. 2007	N.R.
Picarelli et al. 2010	N.R.
Tekin et al. 2014	Sisli Etfal Education And Research Hospital Physical Medicine And Rehabilitation Outpatient Unit
Todorov et al. 2020	N.R.
Umezaki et al. 2016	Medical University Of South Carolina; Emails
Vanhanen et al. 2023	The Helsinki University Hospital Pain Clinic; Private Clinics, And Via Advertisements On Social Media In A Private CRPS Patient Group
YAĞCI et al. 2014	N.R.
**tES protocols**	
**Authors**	***Place of recruitment***
Adhia et al. 2023	Department Of Surgical Sciences Laboratory, Dunedin School Of Medicine, Dunedin Hospital, New Zealand
Alcon et al. 2025	High Point University
Lopes Alves et al. 2024	Hospital De Clınicas De Porto Alegre (Brazil)
Antal et al. 2011	N.R.
Armbrust et al. 2024	School Clinic Of Faculdade Anhanguera De Sorocaba, Sorocaba, Brazil

Arroyo-Fernandez. 2022	Fm Patient Associations
Caumo et al. 2022	Hospital De Clınicas De Porto Alegre (Brazil)
Caumo et al. 2024	Hospital De Clínicas De Porto Alegre (Brazil)
Jornada et al. 2024	Hospital De Clınicas De Porto Alegre (Brazil)
de Melo et al. 2020	N.R.
de Paula et al. 2023	La Salle Saude, Canoasn.R.Rs, Brazil
Dere et al. 2025	Institut Municipal D'investigació́ Mèdica, Barcelona, Spain
Dutra et al. 2020	Natal (Brazil)
Ehsani et al. 2023	N.R.
Fagerlund et al. 2015	Pain Clinic, University Hospital Of Northern Norway, Tromsø
Hasırcı Bayır et al. 2024	Headache Outpatient Clinic
Hazime et al. 2017	University Of São Paulo) And Rehabilitation Centre (Irmandade Santa Casa De Misericordia Of Sao Paulo)
Hervik et al. 2024	Vestfold Hospital Trust In Southern Norway
Hodaj et al.2022	Pain Centre Of The Grenoble Alpes University Hospital
Jiang et al. 2020	University Hospital
Khedr et al. 2017	Assiut University Hospital
Lin et al. 2022	Taipei Medical University Hospital.
Loreti et al. 2023	Municipal Physiotherapy Clinic Of Fátima Do Sul (MPCFS) And Health Units Located In Fátima Do Sul, Mato Grosso Do Sul, Brazil.
Luedtke et al. 2015	N.R.
Matias et al. 2022	Federal University Of Rio Grande Do Norte
Mechsner et al. 2023	The Endometriosis Outpatient Center Of The Charité Universitätsmedizin Berlin, Campus Virchow-Klinikum
Oliveira et al. 2015	Adventist College Of bahia, brazil.
Ramasawmy et al.. 2024	University Medical Center Göttingen, Germany
Riberto et al. 2011	N.R.
Samartin-Veiga et al. 2022	Patients Included In Previous Research, Local Health Centers, Press, Patient Associations
Segal et al. 2021	Lowenstein Rehabilitation Hospital (ra'anana, israel)
Serrano et al. 2022	Hospital De Clínicas De Porto Alegre (HCPA)
Sornkaew et al. 2024	University And The Surrounding Areas
To et al. 2017	The University Hospital antwerp, belgium
Volz et al. 2016	Gastroenterology, Infectious Diseases, Rheumatology

Notes: N.R.. = not reported

Protocol features from the included TMS studies

Table S3. The table summarizes protocol features from TMS studies				
**Study**	**Coil position**	**Sham procedure**	**Side effects**	**Medication**
**Ali AbdElkader et al. 2021**	tangentially over the left DLPFC cortex	Coil placed at 90° to the targeted area	N.R.	N.R.
**Altas et al. 2019**	DLPFC: F3*; vertex: CZ*<br><br>M1: motor hotspot	Reverse positioned coil over vertex (0.1 Hz. %1 RMT)	N.R.	Stable treatment for 4 weeks
**Avery et al. 2015**	Left forehead	Sham coil	N.R.	Allowed
**Badr et al. 2024**	Right DLPFC	Coil perpendicular to the scalp	N.R.	Stable treatment for the last 3 months
**Bilir et al. 2021**	Left DLPFC	Reverse positioned coil	No adverse events reported	Stable treatment for 12 weeks
**Boyer et al. 2014**	Left M1	Sham coil	No side effects	Stable treatment for 4 weeks
**Guinot et al. 2021**	M1 cortex (dominant thenar area)	Sham coil	N.R.	Stable treatment for 12 weeks
**Herrero Babiloni et al. 2024**	M1 of most painful represented painful site	Sham coil	No serious adverse events	Stable pharmacological treatment in the last month
**Joshi et al. 2024**	Left M1 and DLPFC	B-65<br>sham coil surface	No serious adverse events	Stable treatment
**Kankane et al. 2024**	Right DLPFC<br>Left DLPFC	Sham coil	No serious adverse events	N.R.
**Mhalla et al. 2011**	Left M1	Sham coil	Headache, headache lasting <3 hours, dizziness	Stable treatment for 4 weeks
**Passard et al. 2007**	Left M1	Sham coil	N.R.	Stable treatment for 4 weeks
**Picarelli et al. 2010**	M1 contralateral to the painful upper limb	Sham coil	Headache, Neck pain, Dizziness. Generalized seizure, after the seventh rtms session	Stable treatment for 12 weeks
**Tekin et al. 2014**	Left M1	Sham coil	Mild and transient headache	No medications
**Todorov et al. 2020**	M1 and left DLPFC	In Coil placed at 90° to the targeted area	N.R.	N.R.

**Umezaki et al. 2016**	F3*	Sham coil	Headache	Stable treatment for 4 weeks
**Vanhanen et al. 2023**	Secondary somatosensory cortex (S2)	A 75-mm plastic block between the TMS coil and scalp	N.R.	N.R.
**YAĞCI et al. 2014**	Left M1	Coil placed at 90° to the targeted area	Transient headache, daily tinnitus	Stable treatment

* Electrodes montage according to the EEG 10-20 system (Jasper, 1958).
Notes: DLPFC = Dorsolateral Prefrontal Cortex; Hz = Hertz; M1 = primary Motor cortex; mm = millimeters; n.r. = not reported; NSAIDs = Nonsteroidal Anti-Inflammatory Drugs; RMT = Resting Motor Threshold; rTMS = repetitive Transcranial Magnetic Stimulation; S2 = Secondary Somatosensory cortex; TMS = Transcranial Magnetic Stimulation; V1 = primary Visual cortex.

Protocol features from the included tES studies

Table S4. The table summarizes additional protocol features of tES studies included in the meta-analysis.

Study	Target region	Sham procedure	Side effects	Medication
**Adhia et al. 2023**	pgACC, dACC, and S1	the Actisham protocol – Neuroelectrics[1]	No severe adverse effects	Stable treatment throughout the study
**Alcon et al. 2025**	Left DLPFC	30-second ramp up/ ramp down	N.R.	N.R.
**Lopes Alves et al. 2024**	Left M1<br>Left DLPFC	in the initial 30 seconds, in the middle 30 seconds, and in the last 30 seconds of the stimulation	No severe adverse effects	Stable treatment in the last 6 months
**Antal et al. 2011**	Visual Cortex<br>V1	30-second ramp up/ ramp down	Mild tingling sensation, headache, transient mild first-degree burn, decreased appetite, rash, itching, headache with dizziness.	Ongoing treatment allowed
**Armbrust et al. 2024**	M1	the direct current was stopped after 2 min to maintain the illusion that the session began with real direct current	Headache, scalp pain, nausea, tingling, fatigue, skin redness, skin burn, sleepiness, trouble concentrating	Ongoing treatment allowed
**Arroyo-Fernandez et al. 2022**	Left M1	30-second ramp up/ramp down	No severe adverse effects	Ongoing treatment allowed
**Caumo et al. 2022**	Left DLPFC	30-second ramp up/ramp down	Burning sensation, headache, neck pain, mood swings, concentration difficulties	Ongoing treatment allowed
**Caumo et al.2024**	Left M1<br>Left DLPFC	20-second ramp up/ramp down	Mild or moderate side effects	
**Jornada et al. 2024**	Left M1<br>Left DLPFC	30-second ramp up at specific intervals	No severe adverse effects	Stable treatment throughout the study
**de Melo et al. 2020**	Left M1	30-second ramp up/ramp down	No side effects reported	Ongoing treatment allowed
**de Paula et al. 2023**	M1 contralateral to dominant area	30-second ramp up	Tingling, itching, and blushing, Headache, neck ache, scalp pain, burning sensation, sleepiness, and acute mood changes	Stable treatment for 12 weeks

**Dere et al. 2025**	M1	current of 0 mA	No severe adverse effects	No medication allowed during the study
**Dutra et al. 2020**	Left DLPFC	30-second ramp up/ramp down	Skin tingling	N.R.
**Ehsani et al. 2023**	Left DLPFC	Fade-in Short stimulation Fade-out (fissfo) approach	No severe adverse effects	N.R.
**Fagerlund et al. 2015**	Left M1	8-second fade-in period + 30 sec of direct current stimulation + 5-second fade-out	Headache, neck pain, scalp pain, tingling, itching, burning sensations, skin redness, sleepiness, trouble in concentration and acute mood change	Ongoing treatment allowed
**Hasırcı Bayır et al. 2024**	Left DLPFC	30-second ramp up and stimulator turned off	No severe adverse effects	Stable treatment for 3 months
**Hazime et al. 2017**	M1 contralateral to the painful area	30-second ramp up/ramp down	Headache, neck pain, scalp pain, back pain, tingling, itching, redness, burning sensations, sleepiness, trouble concentrating, nausea, mood change	Treatment allowed included analgesics, nsaids, opioids, antidepressants, muscle relaxants but no psychoactive drugs
**Hervik et al. 2024**	Left M1	30-second ramp up/ramp down	No severe adverse effects	Stable treatment throughout the study
**Hodaj, 2022**	Left M1	30-second ramp up/ramp down	No side effects reported	Ongoing treatment allowed
**Jiang et al. 2020**	M1 contralateral to the painful/dominan t area	20-second ramp up/ramp down	Itching, tingling, burning sensation, pain or warmth	Not allowed
**Khedr et al. 2017**	Left M1	30-second ramp up/ramp down	Itching, redness of skin	Itching and redness skin
**Lin et al. 2022**	Left M1	10-second ramp up/ramp down	Headache, neck pain, scalp pain, stinging, itch, burning sensation, drowsiness, concentration difficulties	Ongoing treatment allowed
**Loreti et al. 2023**	Left M1	30-second ramp up/ramp down	Redness at the application site and other mild effects (headache, local tingling, somnolence, local itch, neck ache)	Ongoing treatment allowed
**Luedtke et al. 2015**	Left M1	30-second ramp up/ramp down	Minimal transitory side effects	Ongoing treatment allowed
**Matias et al. 2022**	Left M1	30-second ramp up/ramp down	Headache, tingling, dizziness, nausea	Ongoing treatment allowed
**Mechsner et al. 2023**	M1 dominant hemisphere	30-second ramp up/ramp down	Twitching eyelid, nausea, blurry vision, or flashes of light	Ongoing treatment allowed
**Oliveira et al. 2015**	M1 contralateral to the painful area	30-second ramp up/ramp down	No side effects	Ongoing treatment allowed
**Ramasawmy et al. 2024**	Left M1	17-second ramp-up from 0 mA to 2 mA, 17-second ramp-down to 0.3 mA, 0.3 mA constant current for 19 min 23 seconds,	Redness on the skin under the electrodes, headache	Stable treatment for 4 weeks

		and 3-second ramp down at the end of the session		
**Riberto et al. 2011**	lM1	30-second ramp up/ ramp down	No side effects reported	Ongoing treatment allowed
**Samartin-Veiga et al. 2022**	Left M1<br>Left DLPFC<br>Left OIC	15-second ramp up/ ramp down	No side effects reported	Ongoing treatment allowed
**Segal et al. 2021**	M1 contralateral to painful area	1-minute of ramping up to 1.5 mA and then back to 0 mA	Redness of the skin under the area of the electrode, itching sensation	Ongoing treatment allowed
**Serrano et al, 2022**	left DLPFC	30-second ramp up/ramp down	Mild side effects	Ongoing treatment allowed
**Sornkaew et al. 2024**	M1 (hotspot on the contralateral hemisphere to the painful side)	15-second fade at the beginning of the session	Mild adverse effects of tdcs were reported, including tingling, burning, and itching	Medication influencing neurotransmitters not allowed
**To et al. 2017**	C2 nerve dermatome and bilateral DLPFC	5-second ramp up/ ramp down	No side effects reported	Ongoing treatment allowed
**Volz et al. 2016**	M1 contralateral to painful area	30-second ramp up/ramp down	Headache, Scalp pain, Scalp burning sensation, Tingling, Itching, Skin redness, Sleepiness	Ongoing treatment allowed

Notes: dACC = dorsal Anterior Cingulate Cortex; DLPFC = Dorsolateral Prefrontal Cortex; M1 = primary Motor cortex; mA = milliampere; min = minutes; n.r. = not reported; NSAIDs = Nonsteroidal Anti-Inflammatory Drugs; OIC = Operculo-Insular Cortex; pgACC = pregenual anterior cingulate cortex; S1 = primary Somatosensory cortex; tDCS = transcranial Direct Current Stimulation; V1 = primary Visual cortex.
[1] = see Neri et al **[35]** for a detailed description of Actisham protocol

Therapeutic strategies for NIBS techniques use

Following the definition of Razza and colleagues (2020) [36], we defined the treatment strategy as *monotherapy*, when either TMS or tES is applied as the only treatment (i.e., patients were not treated with other approaches, nor were they taking medication); *add-on* in studies where stimulation was added to the ongoing individual therapy, which was not manipulated nor administered within the study; "*augmentative*" when TMS or tES were applied combined with other experimental treatments (psychological, behavioral or pharmacological).

Considering the augmentative studies, seven studies combined NIBS intervention with physical activity. For instance, Guinot and colleagues (2021) [37] administered rTMS in combination with multimodal training, including physical activity three times a week comprising aerobic training, pool-based exercises, and relaxation.

Others included aerobic exercise sessions [38,39], warm-ups such as cycling or walking, neuromuscular conditioning (strength, localized muscular resistance, and power), neuromotor exercises (agility, coordination, and balance), and cool-down (self-stretching) [40]. Similarly, Dere et al. (2025) [41] combined stabilization exercises with tDCS to treat chronic neck pain. Participants were instructed on neutral postural alignment and taught exercises for cervical chin tuck and craniocervical flexion. These movements were practiced slowly and deliberately across various positions—supine, side-lying, prone, crawling, sitting, and standing. Segal and colleagues (2021) [42] simultaneously administered tDCS and mirror therapy to patients affected by phantom limb. This rehabilitation technique utilizes the reflection of movements from a healthy limb in a mirror to create the illusion of the same movement in a paralyzed or impaired limb, thereby improving residual movement ability or reducing pain [43]. Armbrust et al. (2024) [29], instead of requiring an active movement to patients, combined tDCS with an osteopathic manipulative treatment (OMT) program, in which manipulation was performed by a technician. Hazime et al. (2017) [44] combined real and sham peripheral electrical stimulation (PES) with transcranial direct current stimulation (tDCS) to explore the top-down analgesic effects induced by central (tDCS) and peripheral (PES) neuromodulation techniques. PES is an electrical current delivered by a device that generates a balanced, asymmetric biphasic rectangular waveform, capable of activating a complex neural network involving various neurotransmitters and receptors [45] capable of promoting segmental [46] and extrasegmental [47] analgesia.

Five studies [19,48-51] adopted psychoeducative programs. For instance, Alcon et al. (2025) delivered five sessions of pain neuroscience education, a cognitive-behavioral therapy strategy, which aims to reconceptualize a person's pain experience away from a strict biomedical model of pain and towards a holistic, biopsychosocial model that incorporates all facets of the pain experience. Tilbor et al. (2024) [51] combined dTMS with a pain-directed psychotherapeutic intervention that included audio-guided mindfulness exercises or asking participants to focus directly on physical sensations while recalling recent emotionally distressing events to strengthen the mind-body connection. Similarly, Ramasawmy et al. (2024) [49] combined a home-based tDCS protocol with simultaneously delivered mindfulness meditation. The meditation instructions, recorded by an experienced instructor, emphasized breath awareness and open, non-judgmental observation of thoughts and sensations. Ehsani et al. (2023) [50]

administered tDCS with Cognitive Behavioral Therapy (CBT) focusing on the fear of pain. In the protocol, CBT was delivered in 20-minute sessions twice a week for 4 weeks. CBT was delivered by a pain-management-specialized psychologist. Each session focused on a specific topic, including identifying beliefs about pain and its treatment, reconceptualizing the pain experience, education on pain theories and relaxation techniques, activity scheduling and time-based pacing strategies, cognitive restructuring, training in anger management, and sleep hygiene. Participants received homework after each session to reinforce newly learned skills, along with personalized intersession goals that were collaboratively set with the therapist. Combining physical activity and psychological support benefits, Riberto and colleagues (2011) [19] developed a comprehensive four-month rehabilitation program that included educational interventions and behavioral group therapy focused on pain, physical activity, and occupational therapy. Considering augmentative strategies combining NIBS and pharmacotherapy, de Paula et al. (2023) [52] tested Naltrexone (real or placebo), an analgesic drug, in combination with tDCS for fibromyalgia treatment.

Section C – Quantitative supplementary analysis

This section highlights statistical details considering the primary outcome measures, from model selection to meta-regressions, including moderators (subgroup analyses) and predictors. We defined a-priori clinically relevant moderators and predictors, but we explored them only when they were represented in the studies included in the meta-analysis.

Models' selection

Model comparisons for the primary and secondary outcome measures are provided in Table S5.

Table S5. **Models' selection.**

Model	**df**	**AIC**	**LRT**	**p (LRT)**	**τ2 Level 3**	**τ2 Level 2**
Primary endpoints: Pain intensity pre-post treatment						
**Full model**	**3**	**125.16**			**0.317**	**0.106**
Reduced model	2	129.07	5.91	.015		
Primary endpoints: Emotional distress pre-post treatment						
Full model	3	74.37			0.23	0
**Reduced model**	**2**	**94.16**	**21.79**	**< .001**		
Primary endpoints: Functional disability pre-post treatment						
Full model	3	75.96			0.29	0
**Reduced model**	**2**	**83.22**	**9.26**	**.002**		
Secondary endpoints: Pain intensity pre to follow-up						
Full model	3	45.47			0.10	0
**Reduced model**	**2**	**48.30**	**4.83**	**.028**		
Secondary endpoint: Emotional distress pre to follow-up						
Full model	3	41.98			0.41	0
**Reduced model**	**2**	**51.78**	**11.80**	**< .001**		
Secondary endpoints: Functional disability pre to follow-up						
Full model	3	41.42			0.741	0
**Reduced model**	**2**	**45.52**	**6.10**	**.014**		
Secondary endpoints: Quality of life pre to post						
Full model	3	32.24				
**Reduced model**	**2**	**31.03**	**0.78**	**.376**		

The table summarizes the model selection procedure, comparing the Full model (namely, the three-level regression model) and the reduced model, which excludes the three-level component, for each dependent variable. The reduced model was chosen for all dependent variables but pain intensity pre-post, even though the full model showed a better fit according to AIC and LRT parameters for several measures. Indeed, in those cases, analyses highlighted that all the variance was due to the cluster variance (i.e., between-study level – Level 3).

Notes: df = degrees of freedom; AIC = Akaike's information criterion; LRT = likelihood-ratio test; p = p-value of the LRT; τ2 Level 3 = variance explained at the between-cluster level; τ2 Level 2= variance explained at the within-cluster level.

Primary outcome measures: short-term effects of NIBS on pain intensity

Table S6. Subgroup analysis of the pre-post pain intensity change score.

Moderator / Subgroup analysis	g	SE	LL	UL	t	p	k	$p_{subgroup}$
				Pain intensity pre-post				
NIBS type								.678
**tDCS**	-0.431	0.057	-0.547	-0.314	-7.524	< .001	33	
**rTMS**	-0.820	0.095	-1.023	-0.618	-8.629	< .001	16	
Target region								.265
**lDLPFC**	-1.235	0.305	-1.913	-0.556	-4.053	=.002	11	
**lM1**	-0.458	0.153	-0.779	-0.137	-2.998	= .008	19	
**cpM1**	-0.732	0.203	-1.229	-0.235	-3.603	= .011	7	

The table summarizes the categorical moderators and subgroup analyses. Note: g = Hedges' g effect size; k = the number of effect sizes contributing to g in the same row; LL = lower limit of the 95% CI; p = p-value associated with the t-score in the same row; psubgroup = p-value of the subgroup comparison; SE = standard error of the coefficient; t = t-score associated with the g value in the same row; UL = upper limit of the 95% CI.

Table S7. Covariate analysis of the pre-post pain intensity change score.

Covariate	Estimate	SE	LL	UL	t	p^1	k	Q	p^2
**Illness duration**							32	0.411	.526
**Intercept**	-0.874	0.266	-1.417	-0.331	-3.289	= .003			
**Slope**	0.022	0.034	-0.047	0.090	0.641	= .526			
**Number of sessions**							53	0.524	.472
**Intercept**	-0.783	0.240	-1.265	-0.302	-3.268	= .002			
**Slope**	0.015	0.021	-0.027	0.057	0.724	= .472			
**Number of pulses**							18	0.619	.443
**Intercept**	-1.119	0.545	-2.274	-0.036	-2.055	= .057			
**Slope**	0.001	0.001	-0.001	0.001	0.787	= .443			

The table summarizes the results of continuous moderators. Note: k = number of effect sizes contributing to g in the same row; LL = lower limit of the 95% CI; p^1 = p-value associated with the t-score in the same row; p^2 = p-value of the Q-test for moderation; Q = result of the Q-test for moderation; SE = standard error of the coefficient; t = t-score associated with the g value in the same row; UL = upper limit of the 95% CI.

Considering the moderator (or subgroup) analysis, we could explore the effect of NIBS type (tDCS and rTMS) and the target region (lDLPFC, lM1, cpM1). In Table S6, the p-value in the seventh column shows whether the subgroup-specific effects are significant. Considering the NIBS type, we can see that this is the case for both studies that apply tDCS and rTMS. At the same time, the value under $p_{subgroup}$ shows that the difference in effects between the two subgroups is not significant. The same reasoning can be applied to the brain regions targeted by the included studies: effects were significant in all three subgroups, with no statistical difference among them. Results from the covariates are summarized in Table S7. The three covariates did not predict the studies' effect sizes.

Primary outcome measure: short-term effects of NIBS on emotional distress

Table S8. Subgroup analysis in pre-post emotional distress change score

**Moderator / Subgroup analysis**	**g**	**SE**	**LL**	**UL**	**t**	**p**	**k**	**psubgroup**
**Emotional Distress pre-post**								
**NIBS type**								.062
**tDCS**	-0.213	0.087	-0.391	-0.035	-2.436	= .021	32	
**rTMS**	-0.507	0.131	-0.785	0.230	-3.877	= .001	17	
**Target region**								.310
**lDLPFC**	-0.382	0.195	-0.806	0.042	-1.961	= .074	13	
**lM1**	-0.198	0.082	-0.367	-0.029	-2.412	= .024	26	
**Emotional distress symptoms**								.849
**Anxiety**	-0.328	0.134	-0.608	-0.048	-2.443	=.024	21	
**Depression**	-0.291	0.086	-0.466	-0.115	-3.386	= .002	31	

The table summarizes the categorical moderators and subgroup analyses. Note: g = Hedges' g effect size; k = the number of effect sizes contributing to g in the same row; LL = lower limit of the 95% CI; p = p-value associated with the t-score in the same row; psubgroup = p-value of the subgroup comparison; SE = standard error of the coefficient; t = t-score associated with the g value in the same row; UL = upper limit of the 95% CI.

Table S9. Covariate analysis of the pre-post emotional distress change score

**Covariate**	Estimate	SE	LL	UL	T	p	k	Q	p
**Illness duration**							33	8.370	.007
**Intercept**	-0.704	0.140	-0.990	-0.417	-5.012	< .001			
**Slope**	0.041	0.014	0.012	0.071	2.893	.007			
**Number of sessions**							54	3.724	.059
**Intercept**	-0.037	0.154	-0.346	0.273	-0.237	.814			
**Slope**	-0.024	0.013	-0.050	0.001	-1.930	.059			

The table summarizes the results of continuous moderators. Note: k = number of effect sizes contributing to g in the same row; LL = lower limit of the 95% CI; p^1 = p-value associated with the t-score in the same row; p^2 = p-value of the Q-test for moderation; Q = result of the Q-test for moderation; SE = standard error of the coefficient; t = t-score associated with the g value in the same row; UL = upper limit of the 95% CI.

Considering the moderator (or subgroup) analysis, we could explore the effects of NIBS type (tDCS and rTMS), the target region (lDLPFC and lM1), and emotional distress symptoms, measured by anxiety and depression. In Table S8, the p-values in the seventh column show whether the subgroup-specific effects are significant. Considering the NIBS type, we observe a trend favoring rTMS over tDCS, possibly because rTMS protocols yield larger effect sizes than tDCS protocols. However, caution is warranted when interpreting these results due to the

imbalance in the number of studies comparing tDCS and TMS. Considering the target region and the specific symptoms of emotional distress, no differences emerged in the subgroup analysis. Meta-regressions revealed a significant effect of illness duration: for every additional year of illness, the impact of real stimulation on reducing emotional distress decreases by 0.04, indicating that stimulation is less effective as illness duration increases. The number of sessions showed a trend toward significance in the opposite direction: indeed, for every additional NIBS session, the effect size g in a study is expected to increase by 0.02, suggesting that the impact of real stimulation in reducing emotional distress is more effective.

Primary outcome measure: short-term effects of NIBS on functional disability

Table S10. Subgroup analysis in pre-post functional disability change score

Moderator / Subgroup analysis	g	SE	LL	UL	t	P	k	psubgroup
**Functional Disability pre-post**								
**NIBS type**								.036
**tDCS**	-0.421	0.034	-0.614	-0.229	-4.487	< .001	28	
**rTMS**	-0.886	0.266	-1.488	-0.284	-3.328	.009	10	

The table summarizes the categorical moderator and subgroup analysis. Note: g = Hedges' g effect size; k = the number of effect sizes contributing to g in the same row; LL = lower limit of the 95% CI; p = p-value associated with the t-score in the same row; psubgroup = p-value of the subgroup comparison; SE = standard error of the coefficient; t = t-score associated with the g value in the same row; UL = upper limit of the 95% CI.

Table S11. Subgroup analysis in pre-post functional disability change score

Moderator	Estimate	SE	LL	UL	T	p	k	Q	p
**Illness duration**							20	2.889	.106
**Intercept**	-1.053	0.246	-1.570	-0.536	-4.276	< .001			
**Slope**	0.048	0.028	-0.011	0.108	1.700	.106			
**Number of sessions**							41	0.510	.479
**Intercept**	-0.721	0.263	-1.252	-0.190	-2.747	.009			
**Slope**	0.013	0.019	-0.024	0.051	0.714	.479			

The table summarizes the results of continuous moderators. Note: k = number of effect sizes contributing to g in the same row; LL = lower limit of the 95% CI; p^1 = p-value associated with the t-score in the same row; p^2 = p-value of the Q-test for moderation; Q = result of the Q-test for moderation; SE = standard error of the coefficient; t = t-score associated with the g value in the same row; UL = upper limit of the 95% CI.

Considering the subgroup analysis, we could explore only the effect of NIBS type (tDCS and rTMS). Differences between the two subgroups emerged (p = .036), suggesting that the rTMS effect sizes were larger than those of

tDCS. Regarding emotional distress, caution is warranted when interpreting this result, as the two subgroups differ in the number of effect sizes (28 for tDCS and 10 for rTMS). The explored covariates did not predict the studies' effect sizes (ps > .106) (see Table S11).

Secondary outcome measures: medium-term effects of NIBS on pain intensity

Sixteen studies were included, containing eighteen effect sizes. The reduced model was selected for the analysis. The meta-analysis results are summarized in the forest plot (Figure S1). The random effects model showed a significant effect of real NIBS on pain symptom change score, g = -0.80, 95% CI [-1.23, -0.38], which is different from zero, t = -3.99, p < .001. This result suggests that real stimulation may have a large impact on reducing participants' pain one month after the end of treatment. The meta-analysis also revealed high heterogeneity between studies, $Q_{(17)} = 107.78$, p < .001, $\tau^2 = 0.60$ (SE = 0.26), and $I^2 = 84.23\%$ [73.70; 93.63] (substantial heterogeneity), and PIs [-2.50, 0.89]. The Baujat plot inspection (Figure S2) suggested that the study of Kankane et al. LF (2024) [53] greatly contributed to the heterogeneity of the analysis. The influence analysis confirmed the study as an influential case. The effect size removal, however, only slightly reduced overall significance: g = -0.68, 95% CI [-1.05, -0.31], t = -3.87, p = .001.

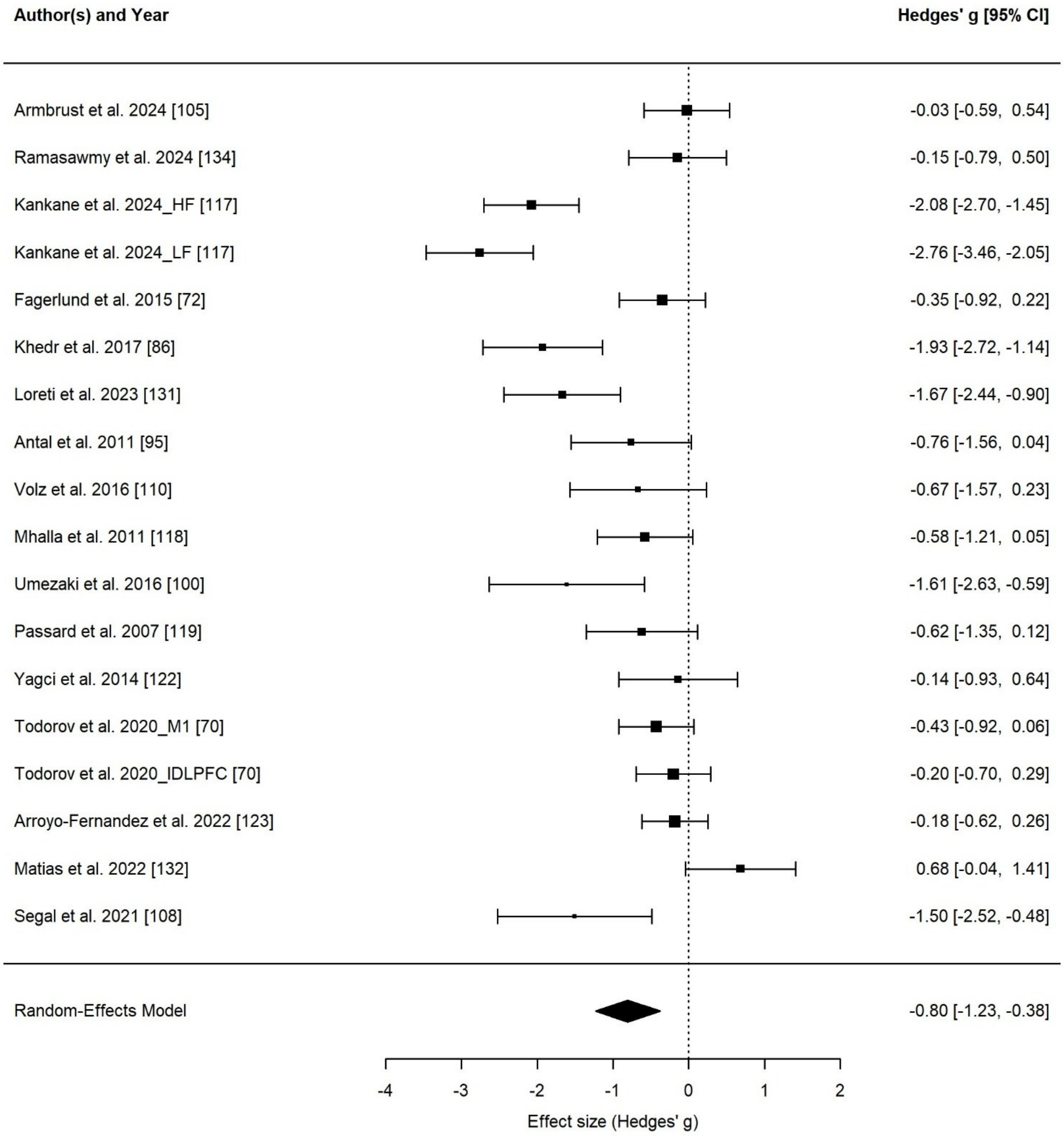

Figure S1. Forest plot of the effect size of NIBS on pain intensity change score at follow-up. CI = confidence interval. Reference numbers refer to the main text bibliography.

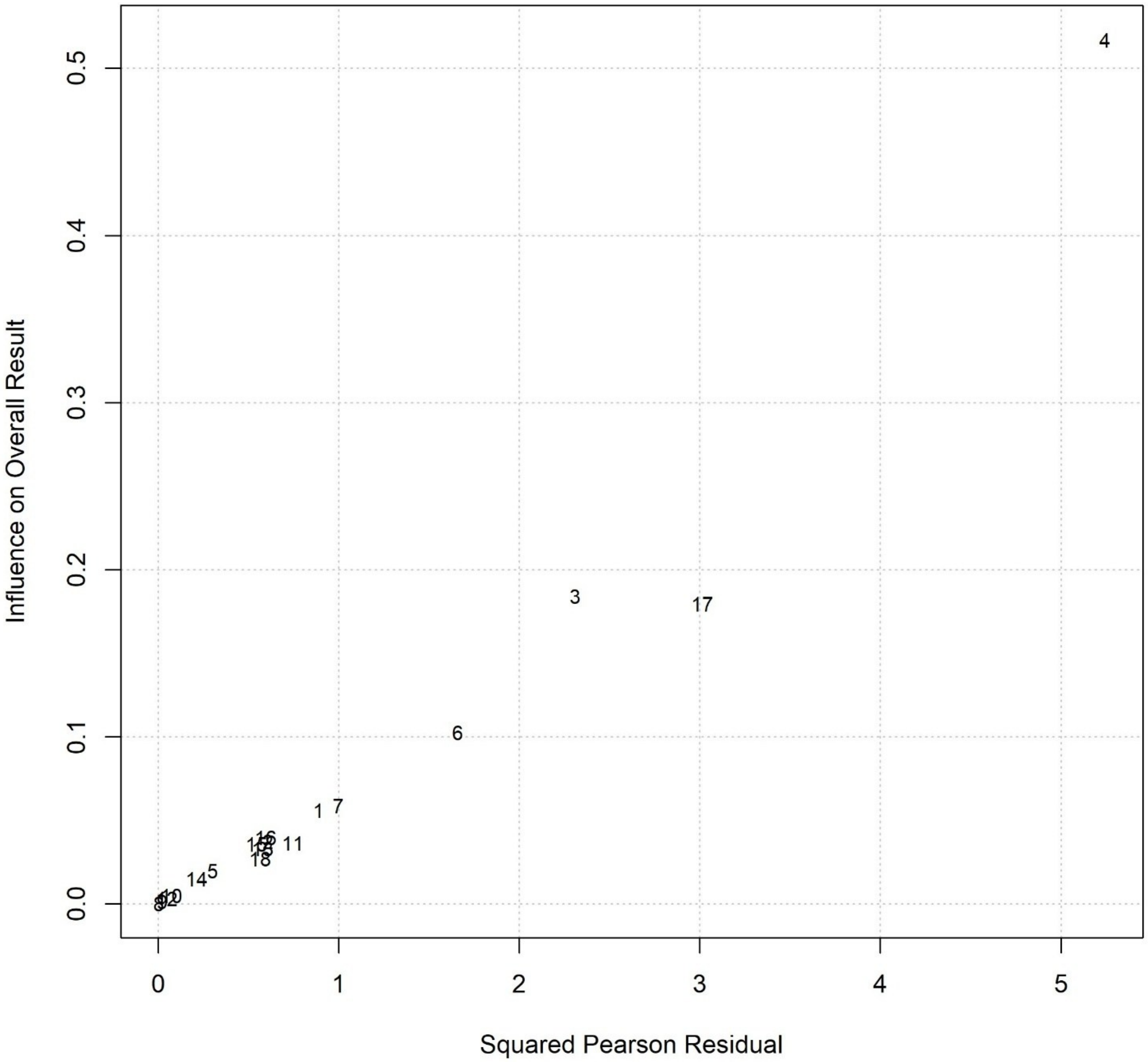

Figure S2. Baujat plot of studies distribution considering pain intensity at follow-up.

**Table S12.** Subgroup analysis of the pre-follow-up pain intensity change score								
**Moderator / Subgroup analysis**	g	SE	LL	UL	t	P	k	psubgroup
**NIBS type**								.326
**tDCS**	-0.609	0.238	-1.148	-0.070	-2.558	.031	10	
**rTMS**	-1.034	0.339	-1.836	-0.233	-3.051	.019	8	

Table S12 summarizes the categorical moderators and subgroup analyses. Note: g = Hedges' g effect size; k = the number of effect sizes contributing to g in the same row; LL = lower limit of the 95% CI; p = p-value associated with the t-score in the same row; psubgroup = p-value of the subgroup comparison; SE = standard error of the coefficient; t = t-score associated with the g value in the same row; UL = upper limit of the 95% CI.

**Table S13.** Meta-regression analysis of the pre-follow-up pain intensity change score									
**Moderator/coefficient**	Estimate	SE	LL	UL	t	p	k	Q	p
**Illness duration**							14	18.681	= .001
**Intercept**	-1.906	0.268	-2.490	-1.329	-7.118	<.001			
**Slope**	0.097	0.022	0.048	0.146	4.322	= 001			
**Number of sessions**							18	2.276	=.151
**Intercept**	-0.050	0.534	-1.182	1.083	-0.093	.926			
**Slope**	-0.085	0.056	-0.203	0.034	-1.509	=.151			

Table S13 summarizes the results of continuous moderators. Note: k = number of effect sizes contributing to g in the same row; LL = lower limit of the 95% CI; p^1 = p-value associated with the t-score in the same row; p^2 = p-value of the Q-test for moderation; Q = result of the Q-test for moderation; SE = standard error of the coefficient; t = t-score associated with the g value in the same row; UL = upper limit of the 95% CI.

In subgroup analyses, we could explore the effects of NIBS type (tDCS and rTMS). Subgroup-specific effects were significant for tDCS and rTMS studies; however, no differences emerged between the two subgroups (p = .851) (Table S12). The analysis of covariates (Table S13) highlights a significant effect of illness duration, accounting for 52.8% of heterogeneity. For every additional year of illness, the effect size (g) of a study is expected to decrease by 0.05. Therefore, we can say that the impact of real stimulation on pain reduction at follow-up is less effective as the years of illness increase, in line with previous studies that suggest that brain modifications are associated with pain duration [54] and may be more resistant to plastic changes induced by NIBS. Even the number of sessions showed a trend toward significance in the opposite direction. Here, for every additional session, the effect size g of a study is expected to increase by 0.07. Therefore, we can say that the impact of real stimulation in our data is augmented by increasing the number of sessions.

Secondary outcome measures: medium-term effects of NIBS on emotional distress

Thirteen studies were included, containing twenty-five effect sizes. The reduced model was chosen.

The meta-analysis results are summarized in the forest plot (Figure S3). The random effects model showed a small effect of real stimulation on the one-month follow-up change score, g = -0.34, 95% CI [-0.57, -0.10], t = -2.94, p = .007. The meta-analysis also revealed substantial heterogeneity between studies, $Q_{(24)} = 78.12$, $p < .001$, $\tau^2 = 0.22$ (SE = 0.10), and $I^2 = 69.28\%$ [57.38; 89.07], and PIs [-1.34, 0.67]. The Baujat plot inspection (Figure S4) suggested that the study by Ehsani et al. (2023) [50] (effect size on anxiety) significantly contributed to the heterogeneity in the analysis, and the influence analysis confirmed it as an influential case. The effect size removal, however, did not change the significance of the results, g = -0.27, 95% CI [-0.47, -0.07], t = -2.79, p = .011. Publication bias using the Pustejovsky-Rodgers' Egger test modification showed no asymmetry: b = -0.30, 95% CI [-4.05, 3.44, t = - 0.04, p = .970.

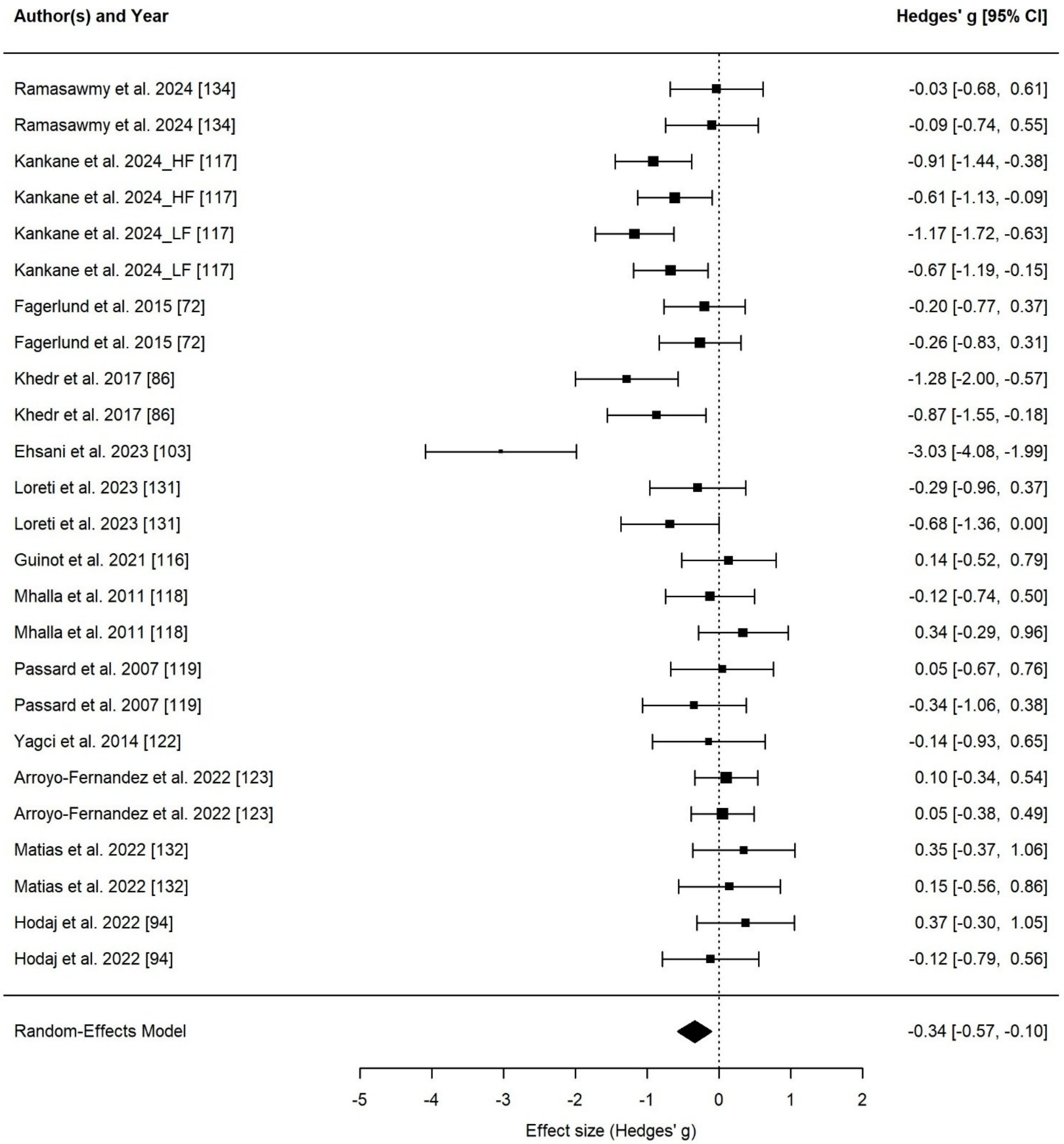

Figure S3. Forest plot of the effect size of NIBS on emotional distress change score at follow-up. CI = confidence interval. Reference numbers refer to the main text bibliography.

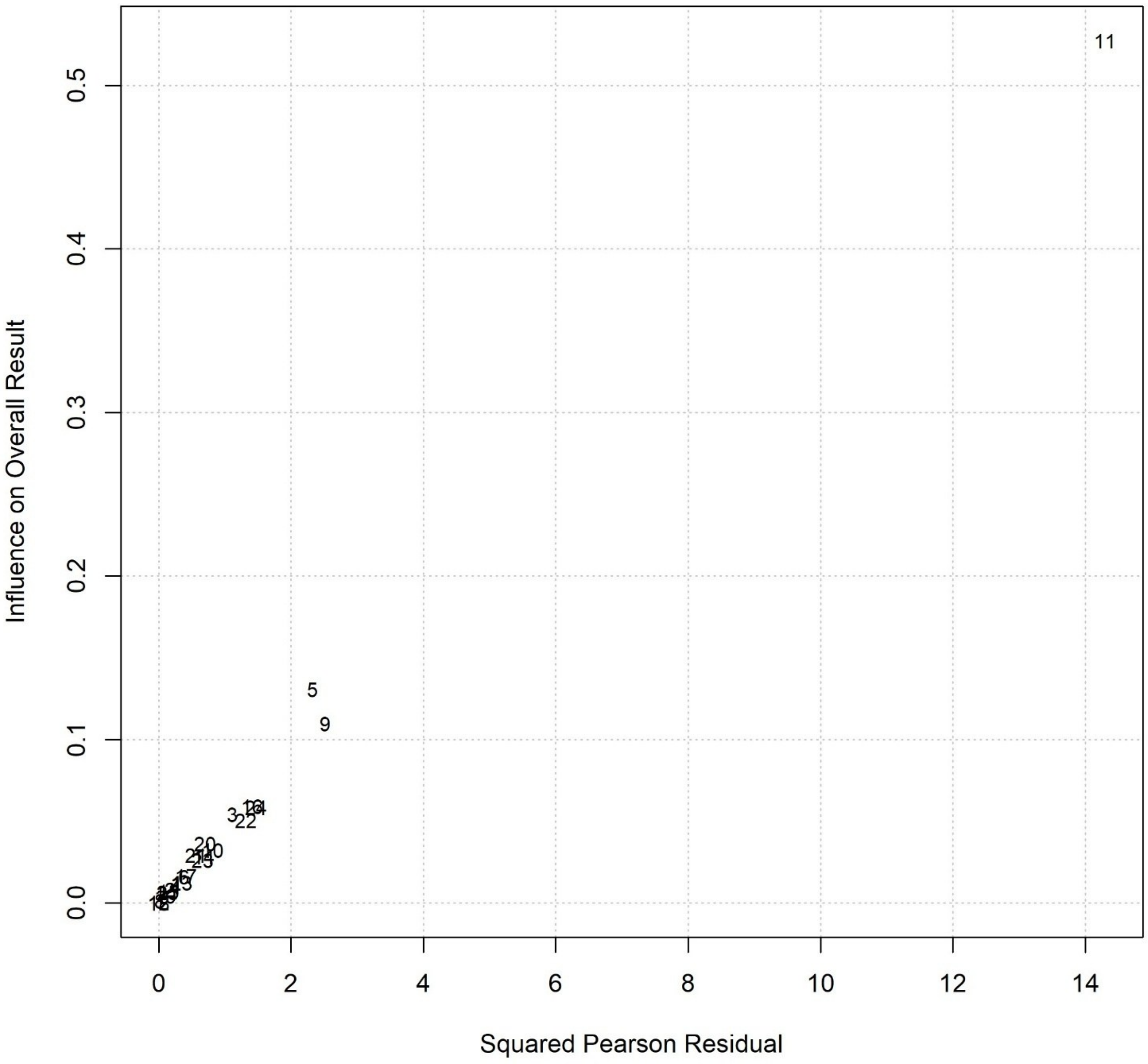

Figure S4. Baujat plot of studies distribution considering emotional distress at follow-up.

Table S14. Subgroup analysis of the pre-follow-up emotional distress change score.

Moderator	g	SE	LL	UL	t	p	k	$p_{subgroup}$
NIBS type								.813
**tDCS**	-0.322	0.160	-0.665	0.022	-2.006	= .065	15	
**rTMS**	- 0.378	0.160	-0.739	- 0.017	-2.368	= .042	10	
Symptoms type								.572
**Anxiety**	-0.437	0.232	-0.948	0.073	-1.885	.086	12	
**Depression**	-0.277	0.135	-0.572	0.018	-2.047	.063	13	

Table S14 summarizes the categorical moderators and subgroup analyses.

Note: g = Hedges' g effect size; k = the number of effect sizes contributing to g in the same row; LL = lower limit of the 95% CI; p = p-value associated with the t-score in the same row; psubgroup = p-value of the subgroup comparison; SE = standard error of the coefficient; t = t-score associated with the g value in the same row; UL = upper limit of the 95% CI.

Table S15. Covariate analysis of the pre-follow-up emotional distress change score.

Covariate	Estimate	SE	LL	UL	t	p	k	Q	p
Illness duration							20	21.317	< .001
**Intercept**	-0.744	0.117	-0.989	-0.499	-6.380	< .001			
**Slope**	0.045	0.010	0.024	0.065	4.617	< .001			
Number of sessions							25	0.006	.937
**Intercept**	-0.308	0.384	-1.103	0.487	-0.803	.430			
**Slope**	-0.003	0.039	-0.085	0.078	-0.080	.937			

Table S15 summarizes the results of continuous moderators.

Note: k = number of effect sizes contributing to g in the same row; LL = lower limit of the 95% CI; p^1 = p-value associated with the t-score in the same row; p^2 = p-value of the Q-test for moderation; Q = result of the Q-test for moderation; SE = standard error of the coefficient; t = t-score associated with the g value in the same row; UL = upper limit of the 95% CI.

Considering the subgroup analysis, we could explore the effect of NIBS type (tDCS and rTMS) and the emotional distress symptoms (anxiety and depression) (Table S14). No specific effects or differences were observed between the moderator subgroups. Covariate analysis highlighted an effect of the illness duration ($p < .001$) (Table S15). For every additional year of illness, the effect size (g) of a study is expected to decrease by 0.05. Therefore, we can say that the impact of real stimulation in reducing emotional distress is less effective when the duration of illness increases.

Secondary outcome measures: medium-term effects of NIBS on functional disability

Fourteen studies were included, containing sixteen effect sizes. The reduced model was chosen.

The meta-analysis results are summarized in the forest plot (Figure S5). The random effects model showed an effect of real NIBS on functional disability change score g = -0.87, 95% CI [-1.36, - 0.38], which is significantly different from zero, t = -3.81, p = .002. This result suggests that real stimulation has a large impact on reducing functional disability, as rated by participants at follow-up. The meta-analysis also revealed substantial heterogeneity between studies, $Q_{(15)} = 118.03$, $p < .001$, $\tau 2 = 0.72$ (SE = 0.32), and I2 = 87.29% [78.79; 95.34], and PIs = [-2.75, 1.01]. Baujat plot inspection (Figure S6) suggested that Kankane et al. 2024 LF [53] and HF, and Loreti et al. 2023 [32] as potential outliers. However, the influence analysis did not highlight influential cases.

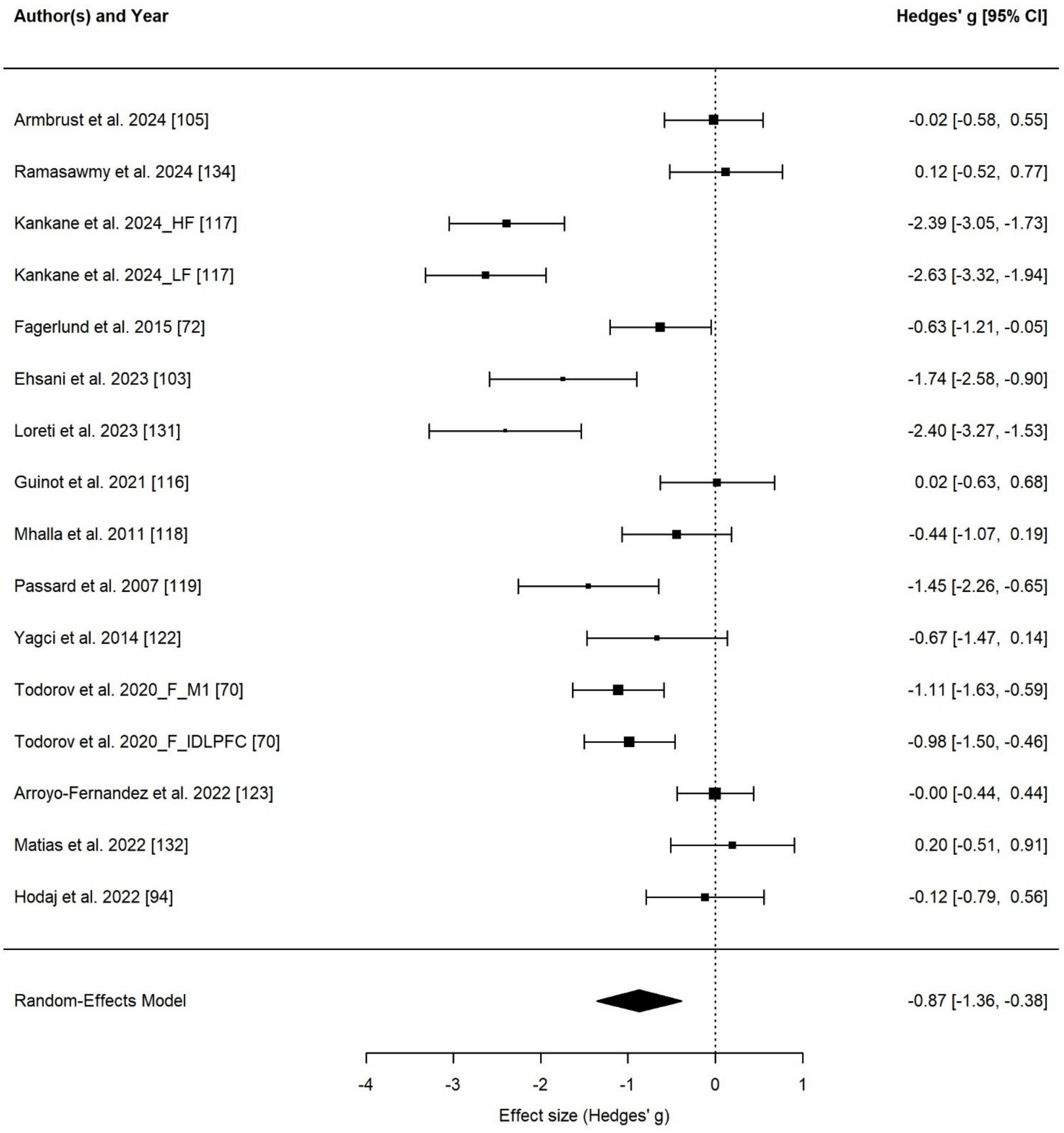

Figure S5. Forest plot of the effect size of NIBS functional disability change score at follow-up. CI = confidence interval. Reference numbers refer to the main text bibliography.

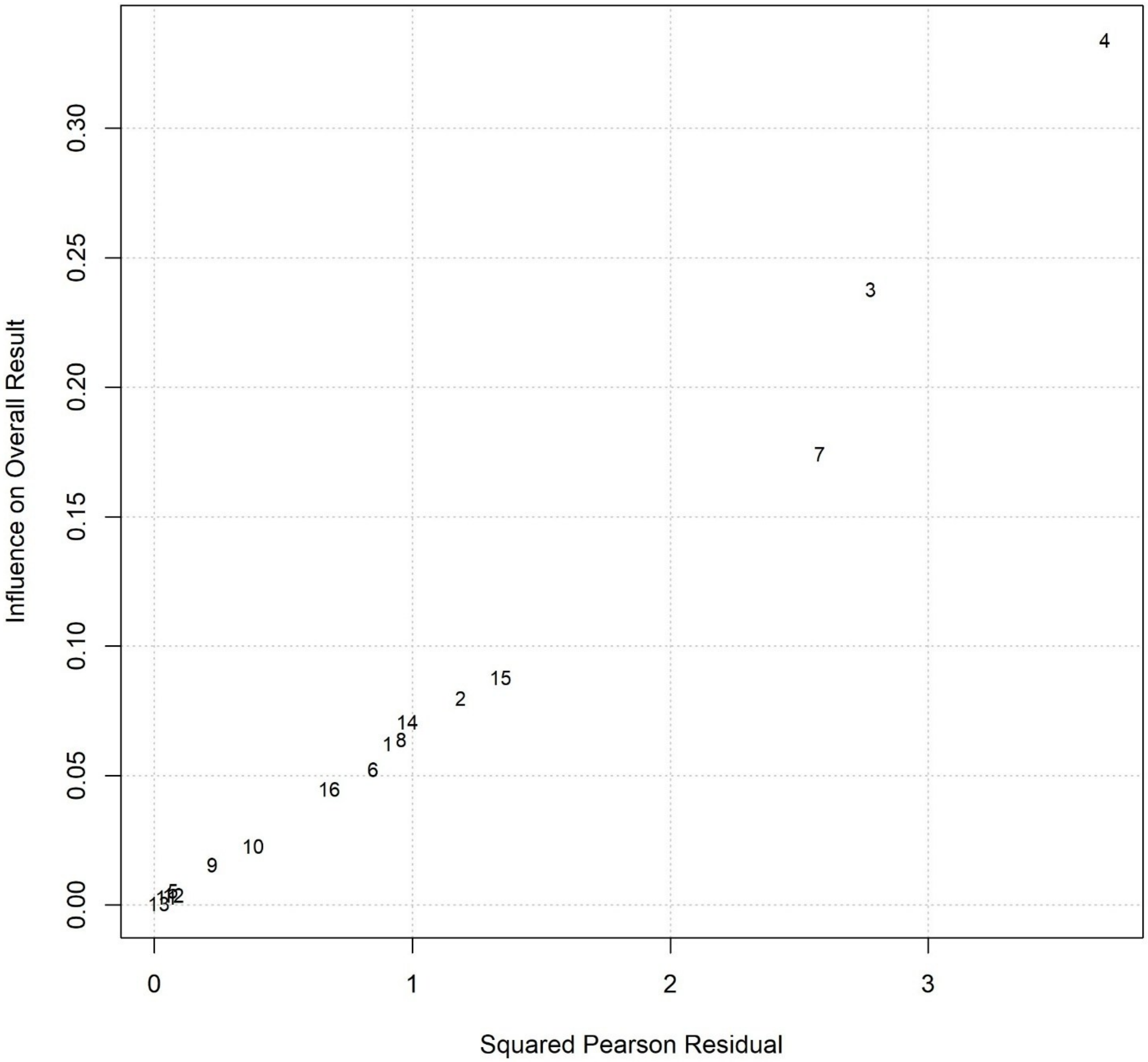

Figure S6. Baujat plot of studies distribution considering functional disability at follow-up.

**Table S16.** Subgroup analysis in pre-follow-up functional disability change score.								
**Moderator**	g	SE	LL	UL	t	p	k	$p_{subgroup}$
NIBS type								.131
**tDCS**	-0.524	0.280	-1.186	0.138	-1.871	.104	8	
**rTMS**	-1.202	0.313	-1.942	- 0.463	-3.844	.006	8	

Table S16 summarizes the categorical moderators and subgroup analyses. Table S16 summarizes the categorical moderators and subgroup analyses.

Note: g = Hedges' g effect size; k = the number of effect sizes contributing to g in the same row; LL = lower limit of the 95% CI; p = p-value associated with the t-score in the same row; psubgroup = p-value of the subgroup comparison; SE = standard error of the coefficient; t = t-score associated with the g value in the same row; UL = upper limit of the 95% CI.

**Table S17.** Subgroup analysis in pre-follow-up functional disability change score.									
**Moderator**	Estimate	SE	LL	UL	t	p^1	k	Q	p^2
Illness duration							12	6.285	.031
**Intercept**	-1.954	0.431	-2.914	-0.994	-4.534	.001			
**Slope**	0.077	0.031	0.009	0.146	2.507	.031			
Number of sessions							16	0.001	.990
**Intercept**	-0.880	0.695	-2.370	0.611	-1.266	.226			
**Slope**	0.001	0.073	-0.156	0.157	0.013	.990			

Table S17 summarizes the results of continuous moderators. Note: k = number of effect sizes contributing to g in the same row; LL = lower limit of the 95% CI; p^1 = p-value associated with the t-score in the same row; p^2 = p-value of the Q-test for moderation; Q = result of the Q-test for moderation; SE = standard error of the coefficient; t = t-score associated with the g value in the same row; UL = upper limit of the 95% CI.

Secondary outcome measures: short-term effects of NIBS on quality of life

Twelve studies were included, containing thirteen effect sizes. The meta-analysis results are summarized in the forest plot (Figure S7). The random effects model did not show an effect of NIBS on the quality-of-life change score *g* = 0.32, 95% CI [-0.08, 0.73], which is not significantly different from zero, t = 1.74, p =.108. This result suggests that real stimulation does not affect the quality of life participants experience after the treatment. Unlike the previous measurements, Hedge's g has a positive value, as standardized questionnaires evaluating quality of life operate in the opposite direction compared to scales measuring previous symptoms; namely, higher scores represent a higher quality of life. The meta-analysis also revealed substantial heterogeneity between studies, $Q_{(12)}$ = 42.89, p < .001, τ2 = 0.31 (SE = 0.20), and I2 = 72.02% [51.24; 92.15], and PIs = [-0.96, 1.61]. The Baujat plot inspection (Figure S8) indicated that the study by Loreti et al. (2023) [32] significantly contributed to the heterogeneity in the statistical analysis. The influence analysis confirmed this study as an influential case. The effect size removal did not change the lack of impact of brain stimulation on quality-of-life scores, g = 0.17, 95%

CI [-0.13, 0.46], $t = 1.26$, $p = .233$. Considering the restricted number of effect sizes included in this analysis, we did not run meta-regression analyses. Moreover, since only three studies included a follow-up measure, we did not conduct analyses on this dependent variable.

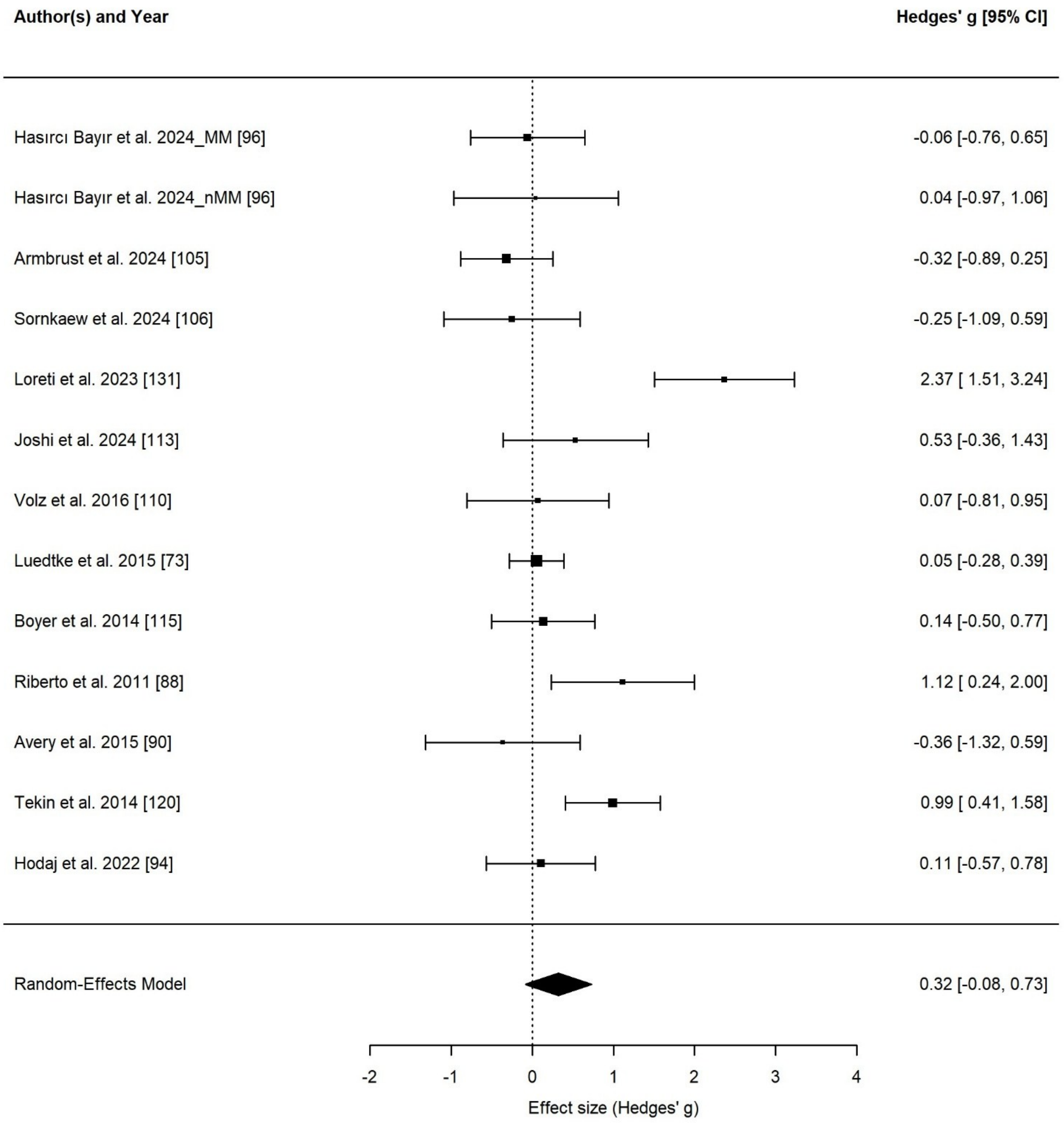

Figure S7. Forest plot of the effect size of noninvasive brain stimulation on quality-of-life change score. Positive effect sizes, in this case, represent a higher quality of life after treatment. CI = confidence interval. Reference numbers refer to the main text bibliography.

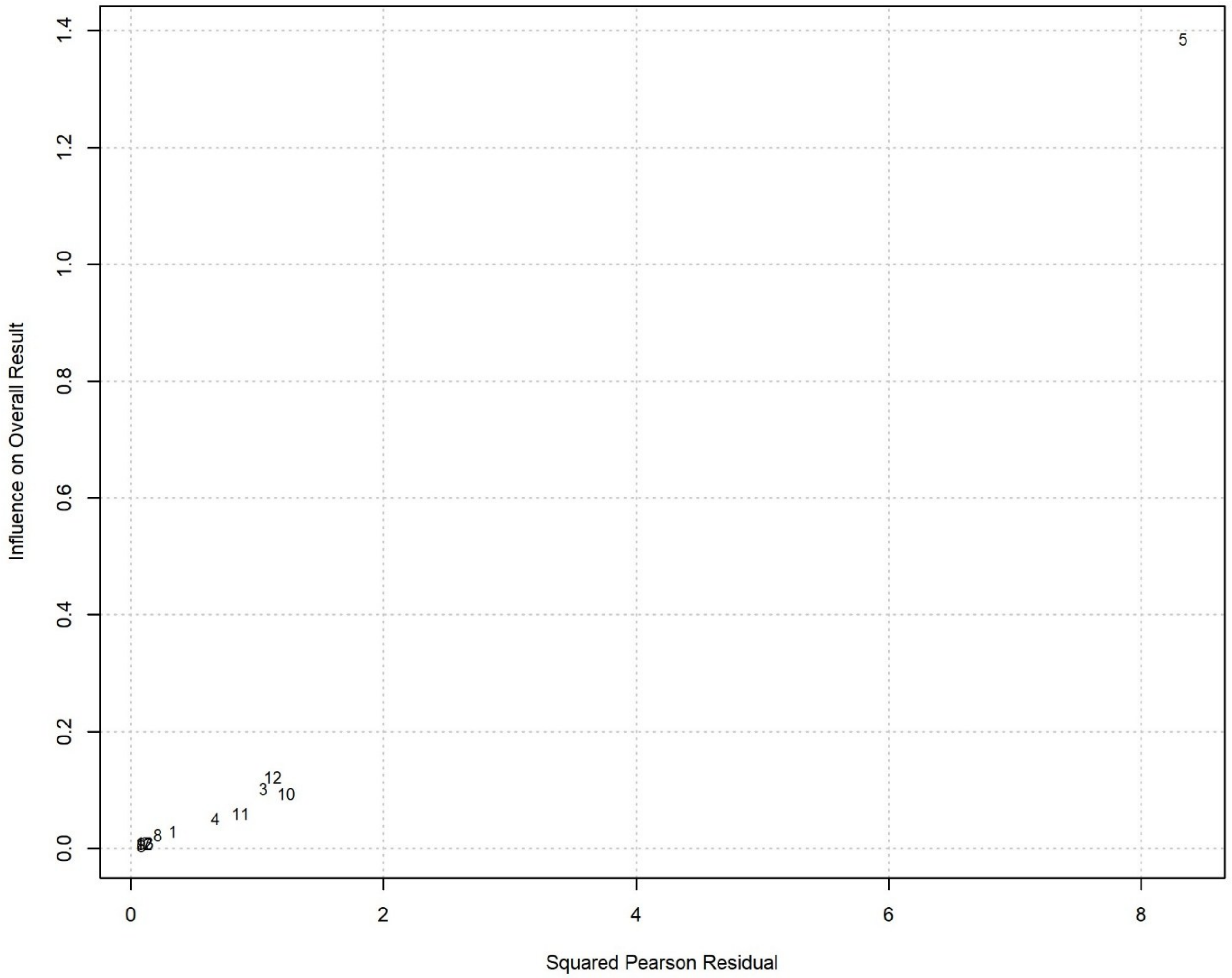

Figure S8. Baujat plot of studies distribution considering quality of life as the outcome measure.

Bibliography

1. Sterne, J.A.; Savović, J.; Page, M.J.; Elbers, R.G.; Blencowe, N.S.; Boutron, I.; Cates, C.J.; Cheng, H.-Y.; Corbett, M.S.; Eldridge, S.M. RoB 2: a revised tool for assessing risk of bias in randomised trials. *bmj* **2019**, *366*.
2. Misra, U.K.; Kalita, J.; Bhoi, S.K. High-rate repetitive transcranial magnetic stimulation in migraine prophylaxis: a randomized, placebo-controlled study. *Journal of neurology* **2013**, *260*, 2793-2801.
3. Hodaj, H.; Payen, J.-F.; Mick, G.; Vercueil, L.; Hodaj, E.; Dumolard, A.; Noëlle, B.; Delon-Martin, C.; Lefaucheur, J.-P. Long-term prophylactic efficacy of transcranial direct current stimulation in chronic migraine. A randomised, patient-assessor blinded, sham-controlled trial. *Brain Stimulation* **2022**, *15*, 441-453.
4. Feng, Y.; Zhang, B.; Zhang, J.; Yin, Y. Effects of Non-invasive Brain Stimulation on Headache Intensity and Frequency of Headache Attacks in Patients With Migraine: A Systematic Review and Meta-Analysis. *Headache: The Journal of Head and Face Pain* **2019**, *59*, 1436-1447, doi:https://doi.org/10.1111/head.13645.
5. Shirahige, L.; Melo, L.; Nogueira, F.; Rocha, S.; Monte-Silva, K. Efficacy of noninvasive brain stimulation on pain control in migraine patients: A systematic review and meta-analysis. *Headache: The Journal of Head and Face Pain* **2016**, *56*, 1565-1596.
6. Spinhoven, P.; Ormel, J.; Sloekers, P.; Kempen, G.; Speckens, A.E.; van Hemert, A.M. A validation study of the Hospital Anxiety and Depression Scale (HADS) in different groups of Dutch subjects. *Psychological medicine* **1997**, *27*, 363-370.
7. Hewitt, P.L.; Norton, G.R. The beck anxiety inventory: a psychometric analysis. *Psychological assessment* **1993**, *5*, 408.
8. Beck, A.T.; Steer, R.A.; Brown, G.K. *Beck depression inventory*; Harcourt Brace Jovanovich New York:: 1987.
9. Hamilton, M. The Hamilton Depression Scale—accelerator or break on antidepressant drug discovery. *Psychiatry* **1960**, *23*, 56-62.
10. Hamilton, M. The assessment of anxiety states by rating. *British journal of medical psychology* **1959**.
11. Leentjens, A.F.; Verhey, F.R.; Lousberg, R.; Spitsbergen, H.; Wilmink, F.W. The validity of the Hamilton and Montgomery-Åsberg depression rating scales as screening and diagnostic tools for depression in Parkinson's disease. *International journal of geriatric psychiatry* **2000**, *15*, 644-649.
12. Spielberger, C.D. State-trait anxiety inventory for adults. **1983**.
13. Roland, M.; Fairbank, J. The Roland–Morris disability questionnaire and the Oswestry disability questionnaire. *Spine* **2000**, *25*, 3115-3124.
14. Nicholas, M.; Vlaeyen, J.W.S.; Rief, W.; Barke, A.; Aziz, Q.; Benoliel, R.; Cohen, M.; Evers, S.; Giamberardino, M.A.; Goebel, A.; et al. The IASP classification of chronic pain for ICD-11: chronic primary pain. *Pain* **2019**, *160*, 28-37, doi:10.1097/j.pain.0000000000001390.
15. Cella, D.F. Quality of life: Concepts and definition. *Journal of Pain and Symptom Management* **1994**, *9*, 186-192, doi:https://doi.org/10.1016/0885-3924(94)90129-5.
16. Felce, D.; Perry, J. Quality of life: Its definition and measurement. *Research in Developmental Disabilities* **1995**, *16*, 51-74, doi:https://doi.org/10.1016/0891-4222(94)00028-8.
17. Ware Jr, J.E. SF-36 health survey update. *Spine* **2000**, *25*, 3130-3139.
18. Avery, D.H.; Zarkowski, P.; Krashin, D.; Rho, W.-k.; Wajdik, C.; Joesch, J.M.; Haynor, D.R.; Buchwald, D.; Roy-Byrne, P. Transcranial magnetic stimulation in the treatment of chronic widespread pain: a randomized, controlled study. *The journal of ECT* **2015**, *31*, 57.
19. Riberto, M.; Alfieri, F.M.; de Benedetto Pacheco, K.M.; Leite, V.D.; Kaihami, H.N.; Fregni, F.; Battistella, L.R. Efficacy of transcranial direct current stimulation coupled with a multidisciplinary rehabilitation program for the treatment of fibromyalgia. *The open rheumatology journal* **2011**, *5*, 45.

20. Boyer, L.; Dousset, A.; Roussel, P.; Dossetto, N.; Cammilleri, S.; Piano, V.; Khalfa, S.; Mundler, O.; Donnet, A.; Guedj, E. rTMS in fibromyalgia: a randomized trial evaluating QoL and its brain metabolic substrate. *Neurology* **2014**, *82*, 1231-1238.
21. Fagerlund, A.J.; Hansen, O.A.; Aslaksen, P.M. Transcranial direct current stimulation as a treatment for patients with fibromyalgia: a randomized controlled trial. *Pain* **2015**, *156*, 62-71.
22. Sornkaew, K.; Thu, K.W.; Silfies, S.P.; Klomjai, W.; Wattananon, P. Effects of combined anodal transcranial direct current stimulation and motor control exercise on cortical topography and muscle activation in individuals with chronic low back pain: A randomized controlled study. *Physiotherapy Research International* **2024**, *29*, doi:10.1002/pri.2111.
23. Hasırcı Bayır, B.R.; Aksu, S.; Gezegen, H.; Karaaslan, Z.; Yüceer, H.; Cerrahoğlu Şirin, T.; Küçükali, C.İ.; Kurt, A.; Karamürsel, S.; Yılmaz, V.; et al. Effects of Transcranial Direct Current Stimulation on Clinical Outcomes, Calcitonin Gene-Related Peptide, and Pituitary Adenylate Cyclase-Activating Polypeptide-38 Levels in Menstrual Migraine. *Neuromodulation: Technology at the Neural Interface* **2024**, *27*, 835-846, doi:https://doi.org/10.1016/j.neurom.2024.01.005.
24. Ware, J.E.; Kosinski, M.; Keller, S.D. A 12-Item Short-Form Health Survey: Construction of Scales and Preliminary Tests of Reliability and Validity. *Medical Care* **1996**, *34*.
25. Luedtke, K.; Rushton, A.; Wright, C.; Jürgens, T.; Polzer, A.; Mueller, G.; May, A. Effectiveness of transcranial direct current stimulation preceding cognitive behavioural management for chronic low back pain: sham controlled double blinded randomised controlled trial. *bmj* **2015**, *350*.
26. Hays, R.D.; Sherbourne, C.D.; Mazel, R.M. The rand 36-item health survey 1.0. *Health economics* **1993**, *2*, 217-227.
27. Volz, M.S.; Farmer, A.; Siegmund, B. Reduction of chronic abdominal pain in patients with inflammatory bowel disease through transcranial direct current stimulation: a randomized controlled trial. *Pain* **2016**, *157*, 429-437.
28. Häuser, W.; Dietz, N.; Grandt, D.; Steder-Neukamm, U.; Janke, K.-H.; Stein, U.; Stallmach, A. Validation of the inflammatory bowel disease questionnaire IBDQ-D, German version, for patients with ileal pouch anal anastomosis for ulcerative colitis. *Zeitschrift für Gastroenterologie* **2004**, *42*, 131-139.
29. Armbrust, D.; Arêas, G.P.T.; Fonseca, C.L.; Arêas, F.Z.d.S.; Duarte, N.d.A.C.; Santana, S.A.A.; Dumont, A.J.L.; Neto, H.P.; Oliveira, C.S. Effects of osteopathic manipulative treatment associated with transcranial direct current stimulation in individuals with chronic low back pain: A double-blind, randomised placebo-controlled trial. *Clinical Rehabilitation* **2024**, *38*, 1609-1621.
30. Santos, M.; Cintra, M.A.; Monteiro, A.L.; Santos, B.; Gusmao-Filho, F.; Andrade, M.V.; Noronha, K.; Cruz, L.N.; Camey, S.; Tura, B. Brazilian valuation of EQ-5D-3L health states: results from a saturation study. *Medical Decision Making* **2016**, *36*, 253-263.
31. Vahedi, S. World Health Organization Quality-of-Life Scale (WHOQOL-BREF): analyses of their item response theory properties based on the graded responses model. *Iranian journal of psychiatry* **2010**, *5*, 140.
32. Loreti, E.H.; Freire, A.M.; Alexandre da Silva, A.; Kakuta, E.; Martins Neto, U.R.; Konkiewitz, E.C. Effects of Anodal Transcranial Direct Current Stimulation on the Primary Motor Cortex in Women With Fibromyalgia: A Randomized, Triple-Blind Clinical Trial. *Neuromodulation: Technology at the Neural Interface* **2023**, *26*, 767-777, doi:https://doi.org/10.1016/j.neurom.2022.11.007.
33. Joshi, S.; Garg, S.; Mishra, P.; Dhyani, M.; Tikka, S.K. Efficacy of sequential primary motor and prefrontal cortices intermittent Theta burst stimulation in persistent somatoform PAIN disorder (TAP-PAIN): A randomized sham-controlled pilot trial. *Indian Journal of Psychiatry* **2024**, *66*, 744-750, doi:10.4103/indianjpsychiatry.indianjpsychiatry_56_24.
34. Tekin, A.; Özdil, E.; Güleken, M.D.; İlişer, R.; Bakım, B.; Öncü, J.; Cevik, M.; Kuran, B. Efficacy of high frequency [10 Hz] repetitive transcranial magnetic stimulation of the primary motor cortex in patients with fibromyalgia syndrome: a randomized, double blind, sham-controlled trial. *Journal of Musculoskeletal Pain* **2014**, *22*, 20-26.
35. Neri, F.; Mencarelli, L.; Menardi, A.; Giovannelli, F.; Rossi, S.; Sprugnoli, G.; Rossi, A.; Pascual-Leone, A.; Salvador, R.; Ruffini, G.; et al. A novel tDCS sham approach based on model-driven controlled shunting. *Brain Stimulation* **2020**, *13*, 507-516, doi:https://doi.org/10.1016/j.brs.2019.11.004.

36. Razza, L.B.; Afonso dos Santos, L.; Borrione, L.; Bellini, H.; Branco, L.C.; Cretaz, E.; Duarte, D.; Ferrão, Y.; Galhardoni, R.; Quevedo, J. Appraising the effectiveness of electrical and magnetic brain stimulation techniques in acute major depressive episodes: an umbrella review of meta-analyses of randomized controlled trials. *Brazilian Journal of Psychiatry* **2020**, *43*, 514-524.
37. Guinot, M.; Maindet, C.; Hodaj, H.; Hodaj, E.; Bachasson, D.; Baillieul, S.; Cracowski, J.-L.; Launois, S. Effects of repetitive transcranial magnetic stimulation and multicomponent therapy in patients with fibromyalgia: a randomized controlled trial. *Arthritis care & research* **2021**, *73*, 449-458.
38. Arroyo-Fernández, R.; Avendaño-Coy, J.; Velasco-Velasco, R.; Palomo-Carrión, R.; Bravo-Esteban, E.; Ferri-Morales, A. Effectiveness of transcranial direct current stimulation combined with exercising in people with fibromyalgia: a randomized sham-controlled clinical trial. *Archives of Physical Medicine and Rehabilitation* **2022**, *103*, 1524-1532.
39. Oliveira, L.; Lopes, T.; Soares, C.; Maluf, R.; Goes, B.; Sá, K.; Baptista, A. Transcranial direct current stimulation and exercises for treatment of chronic temporomandibular disorders: a blind randomised-controlled trial. *Journal of oral rehabilitation* **2015**, *42*, 723-732.
40. Matias, M.G.; Maciel, D.G.; França, I.M.; Cerqueira, M.S.; Silva, T.C.; Okano, A.H.; Pegado, R.; Vieira, W.H.B. Transcranial direct current stimulation associated with functional exercise program for treating fibromyalgia: a randomized controlled trial. *Archives of Physical Medicine and Rehabilitation* **2022**, *103*, 245-254.
41. Dere, T.; Uğur, R.; Ekşi, F.E.; Seyrek, K.; Abakay, H.; Saritaş, N.; Güç, A. Transcranial Direct Current Stimulation Combined with Stabilization Exercises in Chronic Neck Pain: A Randomized Controlled Trial. *Alternative Therapies in Health & Medicine* **2025**, *31*, 36-44.
42. Segal, N.; Pud, D.; Amir, H.; Ratmansky, M.; Kuperman, P.; Honigman, L.; Treister, R. Additive analgesic effect of transcranial direct current stimulation together with mirror therapy for the treatment of phantom pain. *Pain Medicine* **2021**, *22*, 255-265.
43. Ramachandran, V.S.; Rogers-Ramachandran, D.; Cobb, S. Touching the phantom limb. *Nature* **1995**, *377*, 489-490.
44. Hazime, F.; Baptista, A.; De Freitas, D.; Monteiro, R.; Maretto, R.; Hasue, R.; Joao, S. Treating low back pain with combined cerebral and peripheral electrical stimulation: A randomized, double-blind, factorial clinical trial. *European Journal of Pain* **2017**, *21*, 1132-1143.
45. Sluka, K.A.; Walsh, D. Transcutaneous electrical nerve stimulation: Basic science mechanisms and clinical effectiveness. *The Journal of Pain* **2003**, *4*, 109-121, doi:https://doi.org/10.1054/jpai.2003.434.
46. Melzack, R.; Wall, P.D. Pain Mechanisms: A New Theory. *Science* **1965**, *150*, 971-979, doi:doi:10.1126/science.150.3699.971.
47. Kalra, A.; Urban, M.; Sluka, K. Blockade of opioid receptors in rostral ventral medulla prevents antihyperalgesia produced by transcutaneous electrical nerve stimulation (TENS). *The Journal of pharmacology and experimental therapeutics* **2001**, *298*, 257-263.
48. Alcon, C.; Zoch, C.; Luetkenhaus, R.; Lyman, E.; Brizzolara, K.; Goh, H.-T.; Wang-Price, S. Combined transcranial direct current stimulation and pain neuroscience education for chronic low back pain: a randomized controlled trial. *Pain Medicine* **2025**, *26*, 90-97, doi:10.1093/pm/pnae101.
49. Ramasawmy, P.; Gamboa Arana, O.L.; Mai, T.T.; Heim, L.C.; Schumann, S.E.; Fechner, E.; Jiang, Y.; Moschner, O.; Chakalov, I.; Bähr, M.; et al. No add-on therapeutic benefit of at-home anodal tDCS of the primary motor cortex to mindfulness meditation in patients with fibromyalgia. *Clinical Neurophysiology* **2024**, *164*, 168-179, doi:https://doi.org/10.1016/j.clinph.2024.05.018.
50. Ehsani, F.; Hafez Yousefi, M.S.; Jafarzadeh, A.; Zoghi, M.; Jaberzadeh, S. Does Multisession Cathodal Transcranial Direct Current Stimulation of the Left Dorsolateral Prefrontal Cortex Prime the Effects of Cognitive Behavioral Therapy on Fear of Pain, Fear of Movement, and Disability in Patients with Nonspecific Low Back Pain? A. *Brain Sciences* **2023**, *13*, 1381, doi:10.3390/brainsci13101381.
51. Tilbor, E.; Hadar, A.; Portnoy, V.; Ganor, O.; Braw, Y.; Amital, H.; Ablin, J.; Dror, C.; Bloch, Y.; Nitzan, U. TMS in combination with a pain directed intervention for the treatment of fibromyalgia - A randomized, double-blind, sham-controlled trial. *Journal of Psychiatric Research* **2024**, *170*, 167-173, doi:https://doi.org/10.1016/j.jpsychires.2023.12.016.

52. de Paula, T.M.H.; Castro, M.S.; Medeiros, L.F.; Paludo, R.H.; Couto, F.F.; da Costa, T.R.; Fortes, J.P.; de Oliveira Salbego, M.; Behnck, G.S.; de Moura, T.A.M. Association of low-dose naltrexone and transcranial direct current stimulation in fibromyalgia: a randomized, double-blinded, parallel clinical trial. *Brazilian Journal of Anesthesiology (English Edition)* **2023**, *73*, 409-417.
53. Kankane, A.K.; Pandey, A.K.; Patil, M.R.; Agarwal, A. Role of Repetitive Transcranial Magnetic Stimulation in Treatment of Fibromyalgia: A Randomized Controlled Trial. *Annals of Indian Academy of Neurology* **2024**, *27*, 158-164, doi:10.4103/aian.aian_1041_23.
54. Fallon, N.; Brown, C.; Twiddy, H.; Brian, E.; Frank, B.; Nurmikko, T.; Stancak, A. Adverse effects of COVID-19-related lockdown on pain, physical activity and psychological well-being in people with chronic pain. *British Journal of Pain* **2021**, *15*, 357-368, doi:10.1177/2049463720973703.

PRISMA 2020 Checklist

Section and Topic	Item #	Checklist item	Location where item is reported
**TITLE**			
Title	1	Identify the report as a systematic review.	Page 4
**ABSTRACT**			
Abstract	2	See the PRISMA 2020 for Abstracts checklist.	Abstract – Page 4
**INTRODUCTION**			
Rationale	3	Describe the rationale for the review in the context of existing knowledge.	Pages 1-4
Objectives	4	Provide an explicit statement of the objective(s) or question(s) the review addresses.	Page 4
**METHODS**			
Eligibility criteria	5	Specify the inclusion and exclusion criteria for the review and how studies were grouped for the syntheses.	Pages 4-5
Information sources	6	Specify all databases, registers, websites, organisations, reference lists and other sources searched or consulted to identify studies. Specify the date when each source was last searched or consulted.	Page 4 + Supplementary materials Table S1
Search strategy	7	Present the full search strategies for all databases, registers and websites, including any filters and limits used.	Pages 4-5 and supplementary materials Table S1
Selection process	8	Specify the methods used to decide whether a study met the inclusion criteria of the review, including how many reviewers screened each record and each report retrieved, whether they worked independently, and if applicable, details of automation tools used in the process.	Pages 4-5
Data collection process	9	Specify the methods used to collect data from reports, including how many reviewers collected data from each report, whether they worked independently, any processes for obtaining or confirming data from study investigators, and if applicable, details of automation tools used in the process.	Pages 4 -5
Data items	10a	List and define all outcomes for which data were sought. Specify whether all results that were compatible with each outcome domain in each study were sought (e.g. for all measures, time points, analyses), and if not, the methods used to decide which results to collect.	Page 5
	10b	List and define all other variables for which data were sought (e.g. participant and intervention characteristics, funding sources). Describe any assumptions made about any missing or unclear information.	Pages 4-5
Study risk of bias assessment	11	Specify the methods used to assess risk of bias in the included studies, including details of the tool(s) used, how many reviewers assessed each study and whether they worked independently, and if applicable, details of automation tools used in the process.	Page 7-8 + supplementary materials page 1
Effect measures	12	Specify for each outcome the effect measure(s) (e.g. risk ratio, mean difference) used in the synthesis or presentation of results.	Page 5-6
Synthesis methods	13a	Describe the processes used to decide which studies were eligible for each synthesis (e.g., tabulating the study intervention characteristics and comparing against the planned groups for each synthesis (item #5)).	Pages 5-6 +9<br>Supplementary materials pages 3-5
	13b	Describe any methods required to prepare the data for presentation or synthesis, such as handling of missing summary statistics, or data conversions.	Pages 5-6

PRISMA 2020 Checklist

Section and Topic	Item #	Checklist item	Location where item is reported
	13c	Describe any methods used to tabulate or visually display the results of individual studies and syntheses.	Pages 5 - 6
	13d	Describe any methods used to synthesize results and provide a rationale for the choice(s). If meta-analysis was performed, describe the model(s), method(s) to identify the presence and extent of statistical heterogeneity, and software package(s) used.	Pages 5-6
	13e	Describe any methods used to explore possible causes of heterogeneity among study results (e.g. subgroup analysis, meta-regression).	Pages 5-6
	13f	Describe any sensitivity analyses conducted to assess robustness of the synthesized results.	Pages 5-6
Reporting bias assessment	14	Describe any methods used to assess the risk of bias due to missing results in a synthesis (arising from reporting biases).	NA
Certainty assessment	15	Describe any methods used to assess certainty (or confidence) in the body of evidence for an outcome.	Pages 5-6
**RESULTS**			
Study selection	16a	Describe the results of the search and selection process, from the number of records identified in the search to the number of studies included in the review, ideally using a flow diagram.	Pages 6-7
	16b	Cite studies that might appear to meet the inclusion criteria, but which were excluded, and explain why they were excluded.	Pages 6-7
Study characteristics	17	Cite each included study and present its characteristics.	Pages 9-16 Supplementary Materials page 6-11
Risk of bias in studies	18	Present assessments of risk of bias for each included study.	Page 8
esults of individual studies	19	For all outcomes, present, for each study: (a) summary statistics for each group (where appropriate) and (b) an effect estimate and its precision (e.g. confidence/credible interval), ideally using structured tables or plots.	Pages 22-27 and Supplementary Materials Section C
Results of syntheses	20a	For each synthesis, briefly summarise the characteristics and risk of bias among contributing studies.	Pages 22-27 and Supplementary Materials Section C
	20b	Present results of all statistical syntheses conducted. If meta-analysis was done, present for each the summary estimate and its precision (e.g. confidence/credible interval) and measures of statistical heterogeneity. If comparing groups, describe the direction of the effect.	Pages 22-27 and Supplementary Materials Section C
	20c	Present results of all investigations of possible causes of heterogeneity among study results.	Pages 22-27 and Supplementary Materials Section C

PRISMA 2020 Checklist

Section and Topic	Item #	Checklist item	Location where item is reported
	20d	Present the results of all sensitivity analyses conducted to assess the robustness of the synthesized results.	Page 5
Reporting biases	21	Present assessments of risk of bias due to missing results (arising from reporting biases) for each synthesis assessed.	NA
Certainty of evidence	22	Present assessments of certainty (or confidence) in the body of evidence for each outcome assessed.	Pages 22-27 and Supplementary Materials Section C
**DISCUSSION**			
Discussion	23a	Provide a general interpretation of the results in the context of other evidence.	Pages 27-30
	23b	Discuss any limitations of the evidence included in the review.	Pages 30-31
	23c	Discuss any limitations of the review processes used.	Pages 30-31
	23d	Discuss implications of the results for practice, policy, and future research.	Pages 27-31
**OTHER INFORMATION**			
Registration and protocol	24a	Provide registration information for the review, including register name and registration number, or state that the review was not registered.	Page 30-31
	24b	Indicate where the review protocol can be accessed, or state that a protocol was not prepared.	Page 30-31
	24c	Describe and explain any amendments to information provided at registration or in the protocol.	NA
Support	25	Describe sources of financial or non-financial support for the review, and the role of the funders or sponsors in the review.	Page 27
Competing interests	26	Declare any competing interests of review authors.	Page 31
Availability of data, code and other materials	27	Report which of the following are publicly available and where they can be found: template data collection forms; data extracted from included studies; data used for all analyses; analytic code; any other materials used in the review.	Page 31

From: Page MJ, McKenzie JE, Bossuyt PM, Boutron I, Hoffmann TC, Mulrow CD, et al. The PRISMA 2020 statement: an updated guideline for reporting systematic reviews. BMJ 2021;372:n71. doi: 10.1136/bmj.n71.
This work is licensed under CC BY 4.0. To view a copy of this license, visit https://creativecommons.org/licenses/by/4.0/
